# Report of multiple abuse against older adults in three Brazilian cities

**DOI:** 10.1371/journal.pone.0211806

**Published:** 2019-02-08

**Authors:** Rosalina Aparecida Partezani Rodrigues, Ana Maria Ribeiro dos Santos, Maria de Lourdes de Farias Pontes, Edilene Araújo Monteiro, Jack Roberto Silva Fhon, Alisson Fernandes Bolina, Vanessa Costa Almeida, Luipa Michele Silva

**Affiliations:** 1 College of Nursing at Ribeirão Preto, University of São Paulo, Ribeirão Preto, São Paulo, Brazil; 2 Department of Nursing, Federal University of Piauí, Teresina, Piauí, Brazil; 3 Department of Nursing, Federal University of Paraíba, João Pessoa, Paraíba, Brazil; 4 Faculty of Health Sciences, Federal University of Brasília, Federal District, Brasília, Brazil; 5 Department of Nursing, Federal University of Goiás, Catalão, Goiás, Brazil; University of Toronto, CANADA

## Abstract

This study analyses the multiple abuse against older adults reported to the elder abuse police units of three Brazilian cities from 2009 to 2013. This is a longitudinal and retrospective study carried out through the analysis of police reports (PRs) in the elder abuse police units of three Brazilian cities: Ribeirão Preto (SP), Teresina (PI), and João Pessoa (PB). Descriptive statistical analysis consisted of absolute and percentage frequencies. The chi-square test, Fisher’s Exact test, and Relative Risk (RR) were used to analyze the data, with a 95% Confidence Interval (CI) and a significance of 5%. A total of 2,313 reports of older adult abuse were analyzed, of which 245 (10.6%) were related to reports of multiple abuse, 49.4% in Ribeirão Preto, São Paulo, 22.9% in João Pessoa, Paraíba, and 27.8% in Teresina, Piauí. Most of the victims of recurrent older adult abuse are female and younger elderly. Psychological abuse was the most recurrent, followed by financial abuse, occurring in the victim’s own home. In João Pessoa, older women and elderly living with their abusers were at a higher risk of report of multiple abuse acts. In Ribeirão Preto, living with the aggressor was a risk factor for multiple violent acts. In the total population of the study, living without companion and not living with the aggressor were identified as protective factors against recurrent violence. The need to implement social and legal actions to improve safety for the more vulnerable groups is emphasized.

## Introduction

Population ageing is a global phenomenon arising from increasing life expectancy and decreasing fertility. However, current public policies in some countries relegate older adults to a secondary position in relation to young and adult populations. Between 2015 and 2030, the number of people over the age of 60 in the world is predicted to increase by 56%, from 901 million to 1.4 billion. By 2050, this population is predicted to have reached approximately 2.1 billion [1–2]. Between 1980 and 2025, the number of older adults is expected to grow by 217% in Latin America and by 412% in Brazil, where this population will become the sixth largest in the world in absolute numbers [3].

These facts call for social policy changes and pose new challenges to public health management [4]. In this scenario, it is necessary to give greater attention to older adult abuse because this is a multifaceted and multicausal phenomenon with implications in the individual, economic, political and social spheres [5].

International and national studies have highlighted the relevance and magnitude of this problem. A systematic review of 52 studies published between 2002 and 2015, conducted in 28 countries from different regions, found a 15.7% prevalence of older adult abuse in the population studied [6]. The authors also found that in the group aged 60 or over, 11.6% had experienced psychological abuse, 6.8% financial abuse, 4.2% neglect, 2.6% physical abuse, and 0.9% sexual abuse.

An American study addressing the prevalence of older adult abuse in a population of 5,776 older adults found that 715 (12.4%) had experienced one type of violence and 1.7% had experienced more than one. The risk factors identified were: difficulty accomplishing daily-living activities, low social support and past experience of traumatic events [7].

A Brazilian study analyzed 3,593 reports of older adult abuse filed in 2010 in the National Disease Notification System (Sinan Net) in 524 Brazilian towns and cities. The results revealed that 78.8% of the cases occurred at home and 53.6% of the victims reported previous experience of violence. Cases of report of multiple abuse were more common among women than among men [8].

Another study analyzed the characteristics of victims and perpetrators involved in cases of recurrent physical abuse against older adults in five hospitals in Chicago between 2000 and 2011. The study identified that, out of 111 cases of physical abuse, 58 (52.3%) were recurrent. Most cases of physical and sexual violence were observed in the community context, but 15.3% occurred in a nursing home environment. Individuals who were female, widowed, diagnosed with dementia, and still in contact with the perpetrator were substantially more likely to be revictimized [9].

Research has shown that occurrences of older adult abuse, especially at home, are mostly underreported. The victims often avoid reporting the crime for fear of further abuse, which can be considered a risk factor, since they are held hostage by the perpetrator [10].

The analysis of public policy outcomes reveals that older adults frequently seek specialized police stations to report cases of abuse. Criminology research highlights the possibility of report of multiple abuse against groups of older adults, which was also observed in other studies [11–12].

In light of the above, health professionals must be aware of the different forms of older adult abuse, so that they can identify signs of vulnerability and report of multiple abuse and plan intervention actions. Being aware of these characteristics means they can play a fundamental role in recognizing risk factors and, therefore, contributing to abuse prevention.

The objective of the present study was to analyze the report of multiple acts of older adult abuse based on reports filed from 2009 to 2013 in the elder abuse unit of the police department of three Brazilian cities.

## Materials and methods

This longitudinal and retrospective study was carried out through the analysis of police reports (PRs) filed at the elder abuse police unit of three Brazilian cities: Ribeirão Preto (SP), Teresina (PI), and João Pessoa (PB). Data from the Brazilian Institute of Geography and Statistics (IBGE) indicate that Ribeirão Preto is located in the country side of the state of São Paulo, to the northwest of the capital. Its population in 2010 was 604,682 inhabitants, 12.61% of whom were older adults. The other two cities are located in the Northeast Region: Teresina, capital of Piauí, with 814,230 inhabitants, 8.4% of whom were over 60 years old; and João Pessoa, capital of Paraíba, with 723,515 inhabitants, of whom 10.3% were older adults [13].

The Brazilian legislation gives the elderly two possibilities to report complaints of violence: the first is the Elder Abuse Police Unit, and the second is the Dial 100 (in which case the complaint can be anonymous). Both possibilities can be used by the elderly, family members, or third parties who witness the violence. In the case of the second option, the police officer will go to the elderly's home to investigate the case and start the Police Report (PR).

Report of multiple abuse, in this study, is defined by the authors as an abusive act that occurs more than once. “*Ramsey-Klawsnik and Heisler define elder polyvictimization as report of multiple or sequential types of elder abuse by one or more perpetrators*, *or when an older adult experiences one type of abuse perpetrated by multiple people with whom the older adult has a personal*, *professional*, *or caregiver/care recipient relationship in which there is a societal expectation of trust*.*”* ([14] p.15)

### Sample

Reports of multiple older adult abuse (≥ 60 years of age) filed between January 2009 (first year of reports in the police stations) and December 2013 (time when the research project was started) in these cities were analyzed by consulting the PRs, from which data were collected with the aid of an instrument addressing information present in the PRs of potential cases and variables that were relevant to the study.

Recurrence of violence was identified through the cross-referencing of the following information in the PRs: name, gender and birth date of the elderly at the time of the study. Each violent act was registered in separate moments, constituting a new PR. In this article, the report of multiple abuse was treated as the number of violent acts identified in each PR, the type of violence identified, the repetition of multiple events of violence and consequent new visit to the Elder Abuse Police Unit.

### Procedure

The instrument contained two sections. The first section assessed the victim’s and the perpetrator's socio-demographic information in the PR, namely, sex (male and female/categorized), age (full years/categorized in five-year age groups), marital status (unmarried, married, widowed and divorced), schooling (illiterate, elementary school, high school and higher education), and retirement (yes and no). It is worth clarifying that due to the lack of standardization of the filling of the PRs, some information of the victims and aggressors were not registered at the Police Unit.

The second section was the transcription and analysis of the history of violence recorded in the PRs, such as the absolute and average number, and the type of violence and year of recurrence. The cases were reviewed by the all authors of this study and there was a consensus on the number and type of report of multiple abuse acts in the three cities studied.

The project was approved by the Research Ethics Committee of the College of Nursing at Ribeirão Preto—University of São Paulo, protocol nº 36664014.6.1001.5393. The collection of data from the police reports was authorized by the officers responsible for the elder abuse police units in the three cities.

### Statistical analyses

The data was coded and entered into the Statistical Package for Social Sciences (SPSS), version 22.0. The study revealed a gap in socio-demographic information, that is, an incomplete record in the reports regarding data of the older adults and aggressors. Information not available in the reports was therefore considered absent. Descriptive statistical analysis consisted of absolute and percentage frequencies. The chi-square test was used for the categorical variables, the Fisher's Exact test for the categorical variables in which there were data with less than five subjects, being important to identify similarities/differences between the municipalities.

The total number of elderly people in each municipality and in the total population was used obtain the crude Relative Risk (RR), considering recurrence as dependent variable, and gender (male—reference category—and female), age (60 to 79—reference category—and 80 or more); marital status (with partner—reference category—and no partner); retirement (yes—reference category—and no) and cohabitation with the abuser (yes and no—reference category) as independent variables. A 95% Confidence Interval (CI) and significance of 5% were adopted.

## Results

A total of 2,313 reports of elder abuse were analyzed, of which 245 were related to report of multiple acts of abuse. Of these, 49.4% took place in Ribeirão Preto (78.5% had three PRs; 14.9%, four PRs; 5.8%, five PRs; and 0.8%, six PRs), 22.9% in João Pessoa (83.9% had three PRs; and 16.1%, four PRs) and 27.8% in Teresina (82.4% had three PRs; 13.2%, four PRs; and 4.4%, five PRs. The majority of the older adults were female (63.3%), aged 60–69 years, had completed primary education, and were mostly married. The mean age (in years) was 70.60 (± 6.72) in Ribeirão Preto, 70.41 (± 7.53) in João Pessoa, and 69.81 (± 7.91) in Teresina. The median age was 70.0, 69.0 and 69.0 years in Ribeirão Preto, João Pessoa and Teresina, respectively (Table 1).

**Table 1:** Socio-demographic data of older adults involved in cases of report of multiple abuse in three Brazilian cities, 2009–2013. Ribeirão Preto, SP, Brazil.

		Ribeirão Preto		João Pessoa		Teresina		
Study variables	Total	n	%	n	%	n	%	p
**Mean age (= SD); median**		70.6 (6.72); 70.0		70.4 (7.53); 69.0		69.8 (7.91); 69.0		
**Age group (years)**								
60–69	128	60	48.8	32	57.1	37	54.4	0.359†
70–79	81	46	38.0	13	23.2	22	32.4	
80 or more	36	16	13.2	11	19.6	9	13.2	
**Gender**								
Male	90	54	44.6	14	25.0	22	32.4	0.028†
Female	155	67	55.4	42	75.0	46	67.6	
**Marital status**								
Single	31	17	14.2	8	14.8	6	9.0	0.201‡
Married	89	41	34.2	17	31.5	31	46.3	
Widowed	79	38	31.7	18	33.3	23	34.3	
Divorced	36	19	15.8	10	18.5	7	10.4	
Lives with partner	5	5	4.2	0	0.0	0	0.0	
Other	1	0	0.0	1	1.9	0	0.0	
**Level of education**								
Illiterate	32	7	6.0	10	18.5	15	224	<0.001‡
Primary education	144	85	73.3	22	40.7	37	55.2	
Secondary education	34	13	11.2	9	16.7	12	17.9	
Higher education	27	11	9.5	13	24.1	3	4.5	
**Retired**								
No	68	29	25.7	20	39.2	19	29.7	0.214†
Yes	160	84	74.3	31	60.8	45	70.3	

p < 0.05;

Chi-square test†;

Fisher’s exact test‡

Most of the aggressors in the three cities were male, 160 (85.3%), followed by female, 78 (31.8%), and in 7 (2.9%) cases the sex of the aggressor was missing. Age ranged from 15 to 71, with a mean of 38.48 (± 12.26) years. It was also found that 99 (40.4%) were single; 45 (18.4%) married; 20 (8.2%) divorced; 17 (6.9%) lived with the partner; 5 (2.0%) were widowers; and 59 (24.1%) had no information about marital status in the PRs.

It was identified that 141 (57.6%) of the aggressors had family bond with the older adults; 98 (40.0%) did not have this type of bond; and 6 (2.4%) were not identified. Only 109 (44.5%) aggressors lived with the victims.

Regarding the reports of multiple abuse, the total mean of recurrent elder abuse was 2.30 (±0.65) in Ribeirão Preto, 2.16 (±0.37) in João Pessoa and 2.22 (±0.51) in Teresina. The median was 2.00 in the three municipalities, and the 95% CI (Inf-Sup) was 2.18–2.42 in Ribeirão Preto; 2.06–2.26 in João Pessoa; and 2.10–2.34 in Teresina, respectively.

There was more than one form of elder abuse in the total number of cases associated with report of multiple abuse. The most common types of abuse were psychological (51.9%) and financial (25.0%). The majority of cases (80.7%) occurred in the victim's own home (Table 2).

**Table 2:** Type and place of report of multiple abuse according to police reports filed in three Brazilian cities, 2009–2013. Ribeirão Preto, SP, Brazil.

		Ribeirão Preto		João Pessoa		Teresina	
Type of violence	Total	n	%	n	%	n	%
**Psychological**	187	87	46.5	50	26.7	50	26.7
Female		47	54.0	38	76.0	38	76.0
Male		40	46.0	12	24.0	12	24.0
**Financial**	90	24	26.7	19	21.1	47	52.2
Female		15	62.5	14	73.7	28	59.6
Male		9	37.5	5	26.3	19	40.4
**Physical**	71	42	59.2	12	16.9	17	23.9
Female		20	47.6	9	75.0	14	82.4
Male		22	52.3	3	25.0	3	17.6
**Neglect**	8	6	75.0	1	12.5	1	12.5
Female		2	33.3	1	100.0	0	-
Male		4	66.7	0	-	1	100.0
**Abandonment**	4	3	75.0	-	-	1	25.0
Female		2	66.7	0	-	0	-
Male		1	33.3	0	-	1	100.0
**Place of occurrence**							
Residence	197	101	51.3	43	21.8	53	26.9
Public	28	12	41.4	7	24.1	10	34.5
Private	19	8	42.1	6	31.6	5	26.3

In Ribeirão Preto, the year with the highest rate of reports of multiple abuse was 2011 (42.1%). In João Pessoa, this rate reached 41.1% in 2013 and, in Teresina, 30.9% in 2011 and 2012 (Table 3).

**Table 3:** Number of reports of multiple abuse per year and city, 2009–2013. Ribeirão Preto, SP, Brazil.

Year of	Ribeirão Preto		João Pessoa		Teresina	
report	n	%	n	%	n	%
2009	22	18.2	6	10.7	4	5.9
2010	28	23.1	10	17.9	14	20.6
2011	51	42.1	7	12.5	21	30.9
2012	15	12.4	10	17.9	21	30.9
2013	5	4.1	23	41.1	8	11.8

In Ribeirão Preto, it was verified that older adults living with the perpetrator had a higher risk of report of multiple abuse than those who do not live with the perpetrator. On the other hand, in João Pessoa, females had a higher risk of report of multiple abuse when compared with males. There was no statistical significance between the variables regarding the victims of elder abuse in the city of Teresina.

In the total population of the study, living without partner and not living with the aggressor were identified as protective factors against recurrence of violence (Table 4). It is worth mentioning the importance of the quality of the notifications in the years studied (2009–2013), since there was a variation in the frequency of PRs.

**Table 4:** Association between the characteristics of the reports of multiple abuse in three Brazilian cities, 2009–2013. Ribeirão Preto, SP, Brazil.

	Ribeirão Preto		João Pessoa		Teresina		Total Population	
Variables	RR	95% CI	RR	95% CI	RR	95% CI	RR	95% CI
**Gender**								
Female	0.91	0.62–1.34	1.87	1.00–3.49	1.15	0.67–2.00	0.89	0.69–1.14
Age								
80 or more	0.89	0.51–1.56	0.88	0.43–1.78	0.83	0.39–1.76	1.18	0.83–1.66
Marital status								
No partner	1.48	0.99–2.22	1.61	0.89–2.92	1.36	0.77–2.39	0.72	0.56–0.93
Retired								
No	0.69	0.43–1.09	1.03	0.56–1.89	1.39	0.79–2.45	1.09	0.83–1.42
Lives with abuser								
No	1.63	1.10–2.40	0.71	0.36–1.41	1.38	0.80–2.36	0.70	0.55–0.89

p < 0.01; RR: Relative Risk; CI: Confidence Interval

## Discussion

Older adult abuse is a complex issue and a global public health and human rights problem involving socio-demographic and socio-economic aspects [15]. However, research about the forms and incidence of this phenomenon is still in an incipient stage.

The present study found that 10.6% of the cases of older adult abuse in the three cities analyzed were recurrent. This corroborates the findings of a study conducted in the United States, which investigated a sample of cases of older adult maltreatment and found that, in 14% of cases, the maltreatment was ongoing [10]. Likewise, a retrospective analysis of physically abused older patients in five large hospitals in Chicago found that 52.3% had a history of polyvictimization [9].

Research indicates that older adult victims of maltreatment are more susceptible to repeated attacks [9]. A study that used data from the Sinan Net to investigate elder abuse in Brazil found that 53.6% of the victims had been previously abused [8]. A similar result was found in Recife, in the Northeast Region of Brazil, where it was found that 19.42% of older adults of both genders who reported abuse had already experienced a previous situation of maltreatment [16].

The World Health Organization [17] estimates that, globally, 6% of older adults are maltreated and, with the ageing of populations around the world, this type of occurrence is expected to grow. According to a systematic review and meta-analysis conducted in 28 countries, the overall prevalence of abuse was 15.7% in 2017 [6]. Another systematic review found that in North and South America, the prevalence of elder abuse ranged from 10% in cognitively intact older adults to 47.3% in older adults with dementia. In Europe, the prevalence was found to vary between 2.2% and 61.1%. In China, this prevalence was 36.2% and in India 14.0%. In Africa, the prevalence ranged from 30% to 43.7% [15].

Research has shown that older adult abuse has personal consequences, reduces life expectancy, and increases public expenditure [18]. However, because the studies in the literature use different research methodologies and sampling procedures and instruments to evaluate these implications, a variety of risk factors associated with older adult abuse have been observed, which makes it difficult to compare the prevalence rates [15,19].

In the present study, 63.3% of the older adults who reported recurrent abuse were female, 52.2% were between 60 and 69 years old, and the mean age was approximately 70 years. Most were married or widowed, and more than half had completed elementary school. Similar results were found in investigations of cases of recurrent elder abuse in Chicago [9] and Virginia [10]. This is a profile that has been found in several studies analyzing this phenomenon [16,18,20,21,22]. One hypothesis for the greater number of reports from younger older adults is that older adults can find it more difficult to travel to the police station. There is therefore a need for special attention from social workers and health services in order to evaluate the victims and identify cases of report of multiple abuse at home.

Over the years, women have become more independent in society; however, a sexist culture still prevails, so that women are commonly associated with fragility and treated without respect, being often deprived of their ability to control and manage resources, which is especially difficult for older women [21,23–24]. Regarding civil status, an adverse situation was found in this study: widowed, separated, divorced and single older individuals were found to be at greater risk of maltreatment, probably because marriage, over the years, exerts a protective effect in situations of conjugal conflict [8,18,24]. On the other hand, low level of education was identified as a risk factor, as in other studies [16,21,24]. Access to education represents a protective factor for older adults, since it contributes to their independence.

Regarding the place of report of multiple acts of abuse, the majority of cases in this study took place in the victim’s own residence, as was also observed in a study conducted in Chicago [9] and in other studies [8,16,21,24,25]. The home should be the place of greatest security for older adults; however, for many, it represents a space of fear and danger.

As regards the type of abuse, more than half reported psychological abuse, followed by financial abuse. In the analysis of these indicators per city, Ribeirão Preto stood out with a rate of psychological abuse of 46.5%, followed by Teresina with a rate of financial abuse of 52.2%. Likewise, other studies on report of multiple abuse found a prevalence of psychological (22.4%) [9] and financial (53.5%) abuse [10]. These results corroborate other studies on the subject, in which psychological abuse was the most common [6,19,21–22, 24–25], followed by financial abuse [6,22,24].

Research indicates that psychological abuse can trigger feelings of threat and humiliation in older adults, making them vulnerable to other types of abuse [22]. Psychological abuse is considered a trigger for psychological disorders such as anxiety and depression, leading to morbidity and mortality [15]. It is worth noting that financial abuse is usually perpetrated by close relatives, which makes the problem to be underreported [22], especially for three reasons: the victim’s difficulty to report the case; the misinterpretation on the part of the elderly who believe that the abuse was the result of their own lack of ability to manage expenses; and the financial dependence on the abuser [25].

Ribeirão Preto (SP) and João Pessoa (PB) presented a 40% rate of report of multiple abuse, similar to the rates found in a systematic review in European and African countries [15]. It is important to note that there was no pattern in the report rates during the years studied, which suggests that cases have been underreported. This problem has already been mentioned in other studies [9,15, 24– 26], according to which approximately 10% of older adults who suffered violence did not report it to protective services probably because they resided or were in constant contact with the perpetrator and were, therefore, vulnerable to further abuse [10]. Moreover, fear of abandonment is another factor in cases where elders are physically and economically dependent on the perpetrator [9,19].

In this study, residing with the abuser was a risk factor for the report of multiple abuse in the city of Ribeirão Preto. A similar result was found in a systematic review where living with a dysfunctional family represented an important risk factor [27]. Another risk factor for report of multiple abuse was being a woman, as evidenced in other studies that revealed that females were at greater risk of abuse [28–29]. Women are more exposed to who the risk of experiencing emotional or psychological abuse than men [21–24,30].

Given this situation, managers, health professionals, the society and the older adults themselves face the challenge of denouncing cases of abuse. For this to be possible, it is imperative that health professionals be trained to identify these victims in the different services of the health care network and to assist in the creation of public policies to protect the most vulnerable older adults. It is necessary to emphasize the relevance of studies on this phenomenon, which should adopt methodologies that can be applied in different contexts and cultures in order to accurately identify key risk factors and compare prevalence rates. It should be noted that the Brazilian legislation has instituted several important measures to protect the rights of older adults, such as the Federal Constitution, the Statute of the Elderly, the National Policy for the Elderly, and the National Health Policy for the Elderly, but progress is still necessary to ensure that these rights are effectively protected.

### Limitations

The present study found significant results that make it possible to identify relevant factors, with the potential to strengthen public policies in this area. However, it presents three limitations: the type of study, which was based on the analysis of documents that do not follow a standard; the analysis of handwritten reports, which were not always written in a clear and legible way; and the analysis of cases of multiple abuse reported at police stations solely based on the testimony of the older adult who suffered the abuse.

## Conclusion

According to the results of the present study, most of the victims of multiple abuse were female, and younger older adults sought specialized police units more frequently to report this type of crime. Most of the 2,313 PRs analyzed were cases of psychological abuse, mainly in the city of Ribeirão Preto, followed by Teresina and João Pessoa. The risk factors for multiple abuse in the three cities were having a partner and living with the aggressor. The system of legal protection for older adults must adopt protocols for more complete records, and health professionals must be trained to evaluate older adults in order to identify and prevent abuse. A general picture is necessary for planning services or legal/social frameworks, including not just the victim but also the perpetrator. This would allow appropriate services to be planned having families as target. At the same time, society must implement social and legal actions to improve safety for this vulnerable group.

## Supporting information

S1 Dataset Data to elderly. (ZIP)
