# Supplementary material for: Report of multiple abuse against older adults in three Brazilian cities
Source: PLoS One. 2019 Feb 8;14(2):e0211806. doi: 10.1371/journal.pone.0211806 (PMC6368292; doi:10.1371/journal.pone.0211806)
Supplement: S1 Dataset — (ZIP) [file pone.0211806.s001.zip › PB_PARECER_CONSUBSTANCIADO_CEP_1383628.pdf]

ESCOLA DE ENFERMAGEM DE RIBEIRÃO PRETO - USP

PARECER CONSUBSTANCIADO DO CEP

DADOS DO PROJETO DE PESQUISA

Título da Pesquisa: VIOLÊNCIA SOB A PERSPECTIVA DE IDOSOS DA COMUNIDADE
Pesquisador: ROSALINA APARECIDA PARTEZANI RODRIGUES
Área Temática:
Versão: 3
CAAE: 51699515.6.0000.5393
Instituição Proponente: Escola de Enfermagem de Ribeirão Preto - USP
Patrocinador Principal: Financiamento Próprio

DADOS DO PARECER

Número do Parecer: 1.383.628

Apresentação do Projeto:

Trata-se da avaliação das respostas às pendências

Objetivo da Pesquisa:

Sem alterações

Avaliação dos Riscos e Benefícios:

Sem alterações

Comentários e Considerações sobre a Pesquisa:

Sem alterações

Considerações sobre os Termos de apresentação obrigatória:

Sem alterações

Recomendações:

Não há

Conclusões ou Pendências e Lista de Inadequações:

A interessada esclareceu as dúvidas deste CEP, portanto, considero o projeto aprovado.

Considerações Finais a critério do CEP:

Parecer aprovado Ad Referendum.

Este parecer foi elaborado baseado nos documentos abaixo relacionados:

Tipo Documento	Arquivo	Postagem	Autor	Situação
Informações Básicas do Projeto	PB_INFORMAÇÕES_BÁSICAS_DO_PROJETO_623001.pdf	22/12/2015 11:44:47		Aceito
Outros	oficiocepdezembro.pdf	22/12/2015 11:43:20	ROSALINA APARECIDA PARTEZANI RODRIGUES	Aceito
TCLE / Termos de Assentimento / Justificativa de Ausência	TCLE_v3.docx	22/12/2015 11:40:20	ROSALINA APARECIDA PARTEZANI RODRIGUES	Aceito
Projeto Detalhado / Brochura Investigador	Projeto_3.docx	22/12/2015 11:38:38	ROSALINA APARECIDA PARTEZANI RODRIGUES	Aceito
Outros	oficiocep.docx	17/12/2015 17:13:38	ROSALINA APARECIDA PARTEZANI RODRIGUES	Aceito
TCLE / Termos de Assentimento / Justificativa de Ausência	TCLE_nova_versao.docx	17/12/2015 17:08:35	ROSALINA APARECIDA PARTEZANI RODRIGUES	Aceito
Projeto Detalhado / Brochura Investigador	Projeto_com_sugestoes.docx	17/12/2015 17:07:09	ROSALINA APARECIDA PARTEZANI RODRIGUES	Aceito
Outros	oficio.pdf	07/12/2015 22:20:00	ROSALINA APARECIDA PARTEZANI RODRIGUES	Aceito
Projeto Detalhado / Brochura Investigador	Projeto_2.docx	09/11/2015 15:37:28	ROSALINA APARECIDA PARTEZANI RODRIGUES	Aceito
Folha de Rosto	Folha_de_rosto_4.pdf	09/11/2015 15:36:31	ROSALINA APARECIDA PARTEZANI RODRIGUES	Aceito
Orçamento	Orcamento.docx	09/11/2015 09:16:36	ROSALINA APARECIDA PARTEZANI RODRIGUES	Aceito
Cronograma	Proposta_cronograma_de_atividades.doc	09/11/2015 09:14:11	ROSALINA APARECIDA	Aceito

Cronograma	Proposta_cronograma_de_atividades.doc	09/11/2015 09:14:11	PARTEZANI RODRIGUES	Aceito
TCLE / Termos de Assentimento / Justificativa de Ausência	TCLE.docx	09/11/2015 09:05:52	ROSALINA APARECIDA PARTEZANI RODRIGUES	Aceito

Situação do Parecer:
Aprovado

Necessita Apreciação da CONEP:
Não

RIBEIRAO PRETO, 05 de Janeiro de 2016

Assinado por:
Angelita Maria Stabile
(Coordenador)
