# Supplementary material for: Report of multiple abuse against older adults in three Brazilian cities
Source: PLoS One. 2019 Feb 8;14(2):e0211806. doi: 10.1371/journal.pone.0211806 (PMC6368292; doi:10.1371/journal.pone.0211806)
Supplement: S1 Dataset — (ZIP) [file pone.0211806.s001.zip › carta Teresina.pdf]

GOVERNO DO PIAUÍ

GOVERNO DO ESTADO DO PIAUÍ
SECRETARIA DE SEGURANÇA PÚBLICA
POLÍCIA CIVIL JUDICIÁRIA
DELEGACIA GERAL DA POLÍCIA CIVIL
GERÊNCIA DE POLÍCIA ESPECIALIZADA
DELEGACIA DE SEGURANÇA E PROTEÇÃO AO IDOSO – DSPI

DECLARAÇÃO DE AUTORIZAÇÃO

DECLARO, junto a Universidade de São Paulo – USP e Universidade Federal do Piauí-UFPI, para os fins que se fizerem necessários, que autorizo a Profª. Drª. Ana Maria Ribeiro dos Santos a coletar, junto aos arquivos físicos e/ou virtuais desta Delegacia de Segurança e Proteção ao Idoso de Teresina-PI, os dados necessários à instrução da pesquisa intitulada "Violência contra os idosos em dois municípios brasileiros", a ser realizada pelas preditas instituições de ensino superior, objetivando verificar a evolução da ocorrência de violência contra a pessoa idosa, registrada na Delegacia do Idoso de dois municípios brasileiros, sendo um na região sudeste e outro na nordeste, com espaço amostral de 2009 a 2013, cujos dados serão coletados nos Inquéritos Policiais instaurados no sobredito período. Declaro ainda conhecer e cumprir as Resoluções Éticas Brasileiras, em especial a Resolução CNS 466/12 e que esta instituição está ciente de suas co-responsabilidades como instituição co-participante da presente pesquisa e de seu compromisso no resguardo da segurança e bem-estar dos participantes da mesma nela recrutados, dispondo de infra-estrutura necessária para a garantia de tal segurança.

Teresina, 12 de junho de 2014.

Armandino Pinto de Moura
Delegado de Polícia Civil
Titular da Delegacia de Segurança e Proteção ao Idoso
