# Supplementary material for: Report of multiple abuse against older adults in three Brazilian cities
Source: PLoS One. 2019 Feb 8;14(2):e0211806. doi: 10.1371/journal.pone.0211806 (PMC6368292; doi:10.1371/journal.pone.0211806)
Supplement: S1 Dataset — (ZIP) [file pone.0211806.s001.zip › carta RP.pdf]

SECRETARIA DE ESTADO DOS NEGÓCIOS DA SEGURANÇA PÚBLICA
POLÍCIA CIVIL DO ESTADO DE SÃO PAULO
DELEGACIA DE POLÍCIA DE PROTEÇÃO AOS IDOSOS E MEIO AMBIENTE DE RIBEIRÃO PRETO/SP
Rua Goiás, nº 656 – Fone/Fax: (16)3610-6067

DELEGACIA DE POLÍCIA DE PROTEÇÃO AO IDOSO DE RIBEIRÃO PRETO - SP RUA GOIÁS, Nº 656 — CAMPOS ELÍSEOS FONE: (016) 3610-6067

TERMO DE ACEITE

O Senhor Doutor Luiz Geraldo Dias, Delegado de Polícia Titular da Delegacia de Polícia de Defesa do Idoso do Município de Ribeirão Preto-SP, DECLARA que aceita e autoriza a Equipe de Profissionais, Professores Doutores Titulares e Adjuntos ou Assistentes da Escola de Enfermagem de Ribeirão Preto – USP – inclusive seus Doutorandos, a comparecer, frequentar, acessar e pesquisar todos os arquivos de documentos oficiais, registros de boletins de ocorrências, respectivos livros, inclusive, de registros de Inquéritos Policiais, envolvendo toda a sistemática de registros de ocorrências policiais e suas evoluções, tocante a violência contra os idosos, existentes nesta Unidade Policial, para efeito de pesquisas científicas por vários ângulos e áreas, autorizando suas edições de dados em monografias, livros, em papéis ou pelos meios eletrônicos hoje disponíveis para acessamento a todos os profissionais do ramo, da área, tanto quanto, a sociedade brasileira e mundial.

Ribeirão Preto, 24 de julho de 2014

DELEGACIA DE POLÍCIA DE PROTEÇÃO AO IDOSO DE RIBEIRÃO PRETO - SP RUA GOIÁS, Nº 656 — CAMPOS ELÍSEOS FONE: (016) 3610-6067

Luiz Geraldo Dias
Delegado de Polícia Titular.
