# Supplementary material for: Report of multiple abuse against older adults in three Brazilian cities
Source: PLoS One. 2019 Feb 8;14(2):e0211806. doi: 10.1371/journal.pone.0211806 (PMC6368292; doi:10.1371/journal.pone.0211806)
Supplement: S1 Dataset — (ZIP) [file pone.0211806.s001.zip › carta JP.pdf]

DECLARAÇÃO DE AUTORIZAÇÃO DE ATIVIDADES

Pela presente DECLARAÇÃO DE AUTORIZAÇÃO DE ATIVIDADES, eu **Wagner Paiva de Gusmão Dorta**, 1º Superintendente Regional de Polícia Civil do Estado da Paraíba, autorizo a análise dos inquéritos policiais para identificar os tipos de violência sofridas e os encaminhamentos oficiais dos idosos com mais de 60 anos de idade, salientando que a coleta de dados está restrita à Delegacia de Atendimento ao Idoso, na cidade João Pessoa – PB, sob a responsabilidade das Professoras Doutoras Edilene Araújo Monteiro e Maria de Lourdes de Farias Pontes.

João Pessoa, 12 de agosto de 2014

WAGNER PAIVA DE GUSMÃO DORTA
SUPERINTENDENTE DE POLÍCIA CIVIL
