# Supplementary material for: Report of multiple abuse against older adults in three Brazilian cities
Source: PLoS One. 2019 Feb 8;14(2):e0211806. doi: 10.1371/journal.pone.0211806 (PMC6368292; doi:10.1371/journal.pone.0211806)
Supplement: S1 Dataset — (ZIP) [file pone.0211806.s001.zip › BANCO RECORRENCIA VIOLÊNCIA TRÊS MUNICIPIOS.pdf]

NUMQUE	LOCALCOLETA	DAT	BOLETN	ANO	LOCVIO	NRECO	NVEZVIO	SEXOVIT	DATNASVIT
86	1	16/12/2014	157	1	3	66	3	1	27/08/1946
863	1	26/03/2015	11	5	3			1	27/08/1946
864	1	26/03/2015	13	5	3			1	27/08/1946
604	1	12/02/2015	357	3	3	67	2	1	13/04/1945
664	1	11/02/2015	357	3	3			1	13/04/1945
462	1	27/01/2015	489	3	3	1	2	2	15/04/1950
963	1	22/04/2015	401	4	3			2	15/07/1950
454	3	14/09/2015	583	5	3	121	2	1	14/05/1931
555	3	31/07/2015	583	5	1			1	14/05/1931
182	3	02/12/2015	159	3	3	122	2	2	16/06/1945
303	3	04/02/2016	131	4	3			2	16/06/1945
80	1	15/12/2009	2770	1	1	68	2	1	18/11/1933
81	1	15/12/2014	131	1	3			1	18/11/1933
132	3	17/12/2015	2118	3	3	123	2	2	27/03/1942
541	3	22/02/2016	599	5	3			2	27/03/1942
770	1	18/03/2015	110	4	2	69	2	1	12/08/1943
1150	1	04/05/2015	201	5	3			1	12/08/1943
41	3	30/11/2015	2986	2	3	124	2	2	20/03/1942
76	3	14/01/2016	1819	2	3			2	20/03/1942
498	1	29/01/2015	595	3	3	2	2	2	06/01/1943
930	1	15/04/2015	93	5	3			2	06/01/1943
242	2	02/03/2016	65	2	2	188	2	2	13/01/1925
123	2	22/05/2015	65	2	1			2	13/01/1925
556	1	05/02/2015	474	3	3	70	2	1	20/09/1947
631	1	23/02/2015	474	3	3			1	20/09/1947
269	1	15/01/2015	297	2	3	3	2	2	30/09/1936
880	1	26/03/2015	42	5	3			2	30/10/1936
1086	1	04/05/2015	178	5	3	4	2	2	14/03/1926
1166	1	11/05/2015	242	5	3			2	14/03/1926
15	1	09/12/2014	981	1	1	5	2	2	08/09/1941
837	1	24/03/2015	205	4	3			2	08/09/1941
455	3	18/09/2015	561	5	1	125	2	2	19/06/1946
500	3	14/08/2015	561	5	1			2	19/06/1946
295	3	28/01/2016	2175	4	3		2	2	17/06/1938
542	3	22/02/2016	2907	5	3			2	17/06/1938
728	1	16/03/2015	43	4	3	6	2	2	10/03/1940
757	1	16/03/2015	31	4	3			2	10/03/1940
112	3	15/09/2015	152	3	2	126	2	1	28/01/1945
150	3	29/07/2015	152	3	2			1	28/01/1945
969	1	27/04/2015	484	4	3	71	2	1	30/09/1942
97	1	16/12/2014	134	1	2			1	29/08/1936
422	1	26/01/2015	2349	3	3	72	2	1	18/03/1944
968	1	27/04/2015	482	4	3			1	18/03/1944
634	1	23/02/2015	517	3	3	73	2	1	21/03/1949
933	1	15/04/2015	97	5	3			1	21/03/1949
729	1	16/03/2015	45	4	3	74	2	1	12/03/1946
835	1	24/03/2015	201	4	3			1	12/03/1946
235	3	14/09/2015	215	4	1	127	3	1	19/08/2018
321	3	31/07/2015	315	4	1			1	19/08/2018

330	3	25/08/2015	330	4	1			1	19/08/2018
71	3	04/02/2016	255	2	3	128	3	1	07/02/1943
337	3	05/01/2016	442	2	3			1	07/02/1943
338	3	05/01/2016	255	2	3			1	07/02/1943
229	3	21/09/2015	72	4	1	129	2	1	14/08/1944
326	3	01/09/2015	173	4	1			1	14/08/1944
357	1	22/01/2015	44	3	3	75	2	1	14/12/1935
326	1	16/01/2015	64	2	3			1	14/12/1935
73	1	15/12/2014	6942	1	3	76	2	1	02/02/1940
287	1	15/01/2015	27	2	3			1	02/02/1940
934	1	15/04/2015	98	5	3	7	3	2	29/02/1932
967	1	27/04/2015	481	4	3			2	29/02/1932
1141	1	06/05/2015	481	5	3			2	29/02/1932
182	2	16/07/2015	125	2	3	189	2	1	18/11/1942
694	2	30/09/2015	142	5	2			1	18/11/1942
119	1	12/12/2014	3618	1	3	77	2	1	05/07/1940
376	1	22/01/2015	4044	3	3			1	05/07/1940
55	1	11/12/2014	59	1	3	78	2	1	30/08/1927
327	1	16/01/2015	61	2	3			1	30/08/1927
797	2	07/06/2016	72	5	3	190	2	2	01/01/1930
860	2	24/05/2016	73	5	3			2	01/01/1930
292	1	15/01/2015	258	2	3	79	2	1	03/04/1940
805	1	17/03/2015	104	4	3			1	03/04/1940
4	1	09/12/2009	198	1	3	8	3	2	10/06/1933
5	1	09/12/2014	184	1	3			2	10/06/1933
6	1	09/12/2014	189	1	3			2	10/06/1933
228	1	14/01/2015	239	2	3	9	4	2	03/11/1941
484	1	28/01/2015	539	3	3			2	03/11/1941
492	1	29/01/2015	539	3	3			2	03/11/1941
493	1	29/01/2015	619	3	3			2	03/11/1941
670	1	13/02/2015	516	3	3	10	4	2	30/08/1947
673	1	13/02/2015	6832	1	3			2	30/08/1947
675	1	13/02/2015	338	3	3			2	30/08/1947
676	1	13/02/2015	574	3	3			2	30/08/1947
430	1	26/01/2015	128	3	3	80	2	1	07/09/1950
600	1	10/02/2015	4265	3	3			1	07/09/1950
18	3	05/11/2015	1010	1	3	130	2	2	08/12/2028
141	3	17/12/2015	2140	3	3			2	08/12/2028
303	2	10/08/2015	50	3	3	191	3	2	11/12/1942
413	2	14/08/2015	8	4	3			2	11/12/1942
420	2	17/08/2015	15	4	3			2	11/12/1942
580	1	10/02/2015	222	3	3	11	2	2	12/05/1947
1015	1	29/04/2015	428	4	3			2	12/05/1947
374	3	09/03/2016	1209	5	3	131	2	2	27/03/1953
463	3	12/02/2016	482	5	3			2	27/03/1953
232	1	14/01/2015	233	2	3	12	4	2	23/12/1948
586	1	10/02/2015	238	3	3			2	23/12/1948
619	1	20/02/2015	246	3	3	81		1	23/12/1948
620	1	20/02/2015	238	3	3			1	23/12/1948
515	1	30/01/2015	606	3	3	13	3	2	06/09/1934

814	1	18/03/2015	139	4	3			2	06/09/1934
429	1	26/01/2015	135	3	3			2	06/09/1934
697	2	30/09/2015	145	5	1	192	2	2	17/03/1944
672	2	24/09/2015	118	5	1			2	17/03/1944
71	1	16/12/2014	1828	1	3	82	2	1	14/06/1925
82	1	16/12/2014	152	1	3			1	11/06/1925
877	1	26/03/2015	35	5	3	14	2	2	10/09/1943
1123	1	04/05/2015	440	5	3			2	10/09/1943
352	1	21/01/2015	86	3	1	83	2	1	21/02/1947
813	1	18/03/2015	135	4	3			1	21/02/1947
437	1	26/01/2015	167	3	1	15	2	2	16/02/1947
1005	1	06/05/2015	132	5	3			2	16/02/1947
607	1	12/02/2015	380	3	3	16	2	2	15/06/1940
1102	1	06/05/2015	333	5	3			2	15/06/1940
599	1	10/02/2015	253	3	3	17	2	2	16/01/1936
270	1	15/01/2015	296	2	3			2	16/01/1936
399	1	23/01/2015	32	3	3	18	2	2	30/03/1935
400	1	23/01/2015	5869	3	3			2	30/03/1935
588	1	10/02/2015	4045	3	3	19	2	2	06/10/1941
611	1	12/02/2015	4045	3	3			2	06/10/1941
309	1	16/01/2015	1	2	3	84	2	1	31/08/1933
974	1	23/02/2015	452	3	3			1	31/08/1933
505	1	29/01/2015	10054	3	3	85	2	1	18/04/1939
975	1	06/04/2015	327	4	3			1	18/04/1939
431	2	17/08/2015	26	4	1	193	2	2	14/10/1945
545	2	17/06/2016	140	4	3			2	14/10/1945
434	1	26/01/2015	178	3	3	86	2	1	04/07/1944
927	1	15/04/2015	82	5	3			1	04/07/1944
166	1	13/01/2015	120	2	3	87	2	1	
697	1	27/02/2015	120	2	3			1	
175	1	13/01/2015	85	2	2	20	2	2	13/01/1941
755	1	16/03/2015	34	4	3			2	13/01/1941
903	1	04/03/2015	160	2	3	21	2	2	30/03/1930
904	1	04/03/2015	163	2	3			2	30/03/1930
232	3	15/09/2015	157	4	3	132	2	2	27/06/1939
323	3	29/07/2015	58	4	3			2	27/06/1939
608	1	12/02/2015	354	3	3	22	2	2	22/04/1948
662	1	11/02/2015	354	3	3			2	22/04/1948
7	1	09/12/2014	1507	1	3	88	2	1	01/05/1937
28	1	10/12/2014	34	1	3			1	01/05/1937
234	3	21/09/2015	70	4	1	133	2	2	15/03/1952
327	3	27/08/2015	70	4	1			2	15/03/1952
296	3	28/01/2016	1523	4	3	134	2	2	07/07/1943
387	3	18/02/2016	96	5	3			2	07/07/1943
315	3	26/01/2016	2943	4	3	135	2	2	10/04/1951
412	3	12/02/2016	444	5	3			2	10/04/1951
256	3	21/01/2016	67	4	3	136	2	2	19/10/1943
294	3	28/01/2016	2179	4	3			2	19/10/1943
383	3	03/03/2016	683	5	3	137	2	2	20/10/1950
418	3	31/07/2015	559	5	3			2	20/10/1950

32	3	19/11/2015	2308	2	3	138	2	2	17/02/1930
75	3	14/01/2016	734	2	3			2	17/02/1930
164	3	12/01/2016	1704	3	3	139	4	2	18/10/1947
193	3	05/01/2016	1315	3	3			2	18/10/1947
274	3	26/01/2016	954	4	3			2	18/10/1947
282	3	02/02/2016	2316	4	3			2	18/10/1947
90	3	02/12/2015	58	3	3	141	2	1	28/05/1946
465	3	18/09/2015	452	5	1			1	15/02/1953
680	2	24/09/2015	126	5	3	194	2	1	26/09/1935
685	2	22/09/2015	130	5	3			1	26/09/1935
151	2	02/07/2015	94	2	2	195	2	1	18/08/1946
424	2	17/08/2015	19	4	2			1	18/06/1946
11	3	03/11/2015	866	1	2	142	2	1	06/11/1949
26	3	13/10/2015	970	1	2			1	06/11/1949
597	2	26/08/2015	22	5	3	196	2	2	15/09/1944
532	2	03/05/2016	127	4	3			2	15/09/1944
441	1	26/01/2015	479	3	3	23	2	2	30/05/1946
550	1	05/02/2015	398	3	3			2	30/05/1946
44	1	11/12/2014	28	1	1	24	3	2	16/05/1937
138	1	10/12/2014	27	1	1			2	16/05/1937
687	1	25/02/2015	27	1	1			2	16/05/1937
462	2	19/08/2015	57	4	1	197	2	1	20/06/1951
582	2	25/08/2015	8	5	3			1	09/02/1952
131	2	09/06/2015	73	2	3	198	2	1	
362	2	07/03/2016	108	3	3			1	02/02/1942
290	1	15/01/2015	270	2	3	89	2	1	
861	1	26/03/2015	8	5	3			1	05/07/1941
6	2	29/04/2015	5	1	3	199	2	2	15/10/1939
325	2	12/08/2015	72	3	3			2	15/10/1939
815	2	13/06/2016	90	5	3	200	3	2	10/10/1949
10	2	06/05/2015	8	1	3			2	10/10/1949
12	2	06/05/2015	10	1	3			2	10/10/1949
845	2	16/06/2016	121	5	3	201	2	1	10/12/1929
311	2	10/08/2015	58	3	3			1	10/12/1929
824	2	16/06/2016	99	5	3	202	2	2	05/05/1936
867	2	07/06/2016	100	5	3			2	05/05/1936
482	2	21/08/2015	78	4	3	203	3	2	
841	2	16/06/2016	116	5	3			2	18/08/1932
563	2	17/05/2016	159	4	3			2	18/08/1932
425	2	17/08/2015	20	4	3	204	2	2	
872	2	20/06/2016	21	5	3			2	12/10/1937
157	1	12/01/2015	5459	2	3	90	2	1	02/04/1936
243	1	14/01/2015	169	2	2			1	02/04/1936
315	2	12/08/2015	62	3	3	205	2	2	23/02/1934
575	2	24/08/2015	1	5	3			2	23/02/1934
610	1	12/02/2015	367	3	1	25	2	2	01/03/1943
666	1	11/02/2015	367	3	3			2	01/03/1943
143	1	12/01/2015	395	2	3		2	2	22/12/1946
940	1	15/04/2015	372	4	3			2	22/12/1946
170	1	13/01/2015	197	2	3	91	4	1	24/10/1942

358	1	22/01/2015	25	3	3			1	24/10/1942
544	1	04/02/2015	417	3	3			1	24/10/1942
795	1	20/03/2015	181	4	1			1	24/10/1942
351	1	21/01/2015	91	3	3	92	6	1	15/10/1934
445	1	27/01/2015	589	3	3			1	15/10/1934
622	1	20/02/2015	91	3	3			1	15/10/1934
623	1	20/02/2015	589	3	3			1	15/10/1934
824	1	24/03/2015	256	4	3			1	15/10/1934
1180	1	25/05/2015	15091	5	3			1	15/10/1934
787	1	20/03/2015	2627	4	3	93	2	1	12/08/1928
956	1	15/04/2015	389	4	3			1	12/08/1928
113	3	15/09/2015	777	3	3	143	2	1	01/02/1939
152	3	29/07/2015	977	3	3			1	01/02/1939
224	1	14/01/2015	195	2	2	94	2	1	24/10/1949
368	1	22/01/2015	3	3	1			1	24/10/1949
578	2	25/08/2015	4	5	3	206	2	1	10/06/1943
599	2	26/08/2015	24	5	3			1	10/06/1943
658	2	09/09/2015	103	5	3	207	2	1	18/02/1940
669	2	09/09/2015	113	5	3			1	18/02/1940
451	1	27/01/2015	495	3	1	95	2	1	05/11/1939
452	1	27/01/2015	495	3	1			1	05/11/1939
25	1	10/12/2014	5314	1	3	96	2	1	20/05/1925
489	1	29/01/2015	10114	3	3			1	20/05/1925
259	3	19/01/2016	220	4	3	144	2	1	09/07/1941
414	3	12/02/2016	708	5	3			1	09/07/1941
391	3	24/02/2016	1247	5	3	145	2	1	16/11/1945
392	3	09/03/2016	494	5	3			1	16/11/1945
60	3	06/11/2015	74	2	1	146	2	1	20/09/1947
534	3	19/08/2015	788	5	1			1	20/09/1947
625	1	23/02/2015	2	1	3	97	3	1	08/07/1948
431	1	26/01/2015	126	3	3			1	08/07/1948
35	1	10/12/2009	2	1	3			1	08/07/1948
262	3	14/01/2016	28	4	3	147	2	1	16/04/1932
395	3	01/03/2016	949	5	3			1	26/06/1935
504	3	08/10/2015	311	5	1	148	2	1	20/06/1950
365	3	11/09/2015	193	5	3			1	08/08/1936
349	1	21/01/2015	90	3	3	98	2	1	10/05/1949
745	1	16/03/2015	62	4	3			1	10/05/1949
403	2	24/03/2016	146	3	3	208	2	1	09/07/1944
568	2	23/05/2016	165	4	3			1	09/07/1944
843	1	24/03/2015	216	4	3	99	2	1	25/09/1940
1023	1	29/04/2015	437	4	3			1	25/09/1940
183	1	13/01/2015	3783	2	2	100	2	1	11/05/1943
1062	1	22/04/2015	411	4	3			1	11/05/1943
379	2	30/03/2016	124	3	2	209	2	2	08/12/1938
386	2	01/04/2016	130	3	2			2	08/12/1938
105	3	12/01/2016	253	3	3	149	2	2	03/07/1933
228	3	04/02/2016	33	4	3			2	03/07/1933
707	2	16/10/2015	155	5	3	210	2	2	12/12/1950
479	2	20/08/2015	75	4	3			2	12/12/1950

323	2	12/08/2015	70	3	3	211	2	1	24/05/1951
656	2	04/09/2015	101	5	1			1	24/05/1951
58	2	05/06/2015	1	2	3	212	2	2	31/07/1925
75	2	15/06/2015	18	2	3			2	31/07/1925
192	3	22/12/2015	1264	3	3	150	2	1	01/07/1939
404	3	02/03/2016	1064	5	3			1	01/07/1939
250	1	14/01/2015	179	2	3	26	2	2	22/06/1944
915	1	13/04/2015	289	4	3			2	22/06/1944
320	1	16/01/2015	7	2	3	101	2	1	29/09/1936
688	1	25/02/2015	82	1	3			1	29/09/1936
92	1	16/12/2014	172	1	3	102	3	1	06/12/1939
706	1	27/02/2015	172	1	3			1	06/12/1939
707	1	27/02/2015	123	2	3			1	06/12/1939
499	2	24/08/2015	95	4	3	213	2	2	12/07/1942
550	2	17/06/2016	145	4	3			2	12/07/1942
850	1	24/03/2015	230	4	3	103	2	1	01/06/1927
922	1	13/04/2015	4551	4	3			1	01/06/1927
94	1	16/12/2014	5388	1	2	27	2	2	18/03/1940
95	1	16/12/2014	6945	1	3			2	18/03/1940
286	1	15/01/2015	29	2	3	28	2	2	25/05/1940
476	1	28/01/2015	536	3	3			2	25/05/1940
997	1	28/04/2015	341	4	2	29	2	2	04/12/1939
1162	1	11/05/2015	231	5	2			2	04/12/1939
22	2	18/05/2015	21	1	3	214	3	1	05/03/1949
661	2	09/09/2015	106	5	3			1	
133	2	09/06/2015	76	2	3			1	
94	3	03/08/2015	697	3	3	151	2	1	21/06/1934
124	3	29/07/2015	697	3	3			1	21/06/1934
217	3	31/07/2015	436	4	3	152	2	2	12/05/1951
405	3	02/03/2016	436	5	3			2	12/05/1951
96	3	08/09/2015	95	3	3	153	2	2	05/01/1950
549	3	03/03/2016	634	5	3			2	05/01/1950
225	1	14/01/2015	199	2	3	30	2	2	12/12/1946
514	1	30/01/2015	581	3	3			2	12/12/1946
176	1	13/01/2015	82	2	1	31	3	2	16/05/1938
1007	1	06/05/2015	125	5	3			2	16/05/1938
1031	1	29/04/2015	525	4	3			2	16/05/1938
835	2	16/06/2016	110	5	3	215	2	2	17/07/1947
359	2	11/03/2016	105	3	3			2	17/07/1947
1075	1	27/04/2015	503	4	1	32	2	2	30/03/1939
1081	1	04/05/2015	166	5	3			2	30/03/1939
249	3	03/08/2015	673	4	3	154	3	1	04/03/2019
357	3	10/08/2015	1062	5	3			1	04/03/2019
520	3	22/09/2015	834	5	1			1	04/03/2019
178	3	23/12/2015	116	3	3	155	2	1	13/12/1943
247	3	03/08/2015	458	4	3			1	13/12/1943
822	1	24/03/2015	3784	4	3	33	2	2	15/09/1945
823	1	24/03/2015	251	4	1			2	15/09/1945
269	3	05/02/2016	694	4	3	156	2	2	05/09/1947
499	3	10/03/2016	1070	5	3			2	05/09/1947

142	3	15/12/2015	336	3	3	157	2	2	06/10/1940
174	3	15/12/2015	290	3	3			2	06/10/1940
438	1	26/01/2015	492	3	3	34	2	2	22/11/1945
1011	1	06/05/2015	136	5	3			2	22/11/1946
510	1	30/01/2015	1302	3	3	35	2	2	05/01/1946
972	1	27/04/2015	490	4	3			2	02/06/1950
551	1	05/02/2015	405	3	3	36	2	2	17/07/1943
682	1	25/02/2015	405	3	3			2	17/07/1943
409	2	14/08/2015	4	4	3	216	2	2	21/03/1942
606	2	28/08/2015	31	5	3			2	21/03/1942
747	2	02/06/2016	19	5	3	217	2	2	
530	2	03/05/2016	125	4	3			2	17/01/1940
180	3	02/12/2015	31	3	3	158	2	2	08/02/1945
258	3	21/01/2016	233	4	3			2	08/02/1945
598	1	10/02/2015	254	3	3	37	2	2	18/08/1930
885	1	31/03/2015	56	5	3			2	18/08/1930
86	3	20/11/2015	2005	2	3	159	2	2	04/02/1949
300	3	11/02/2016	417	4	3			2	04/02/1949
653	2	04/09/2015	98	5	1	218	2	2	08/08/1938
329	2	12/08/2015	76	3	2			2	08/08/1938
396	1	23/01/2015	1821	3	3	38	3	2	05/01/1946
507	1	29/01/2015	624	3	2			2	05/01/1946
616	1	20/02/2015	506	3	3			2	05/01/1946
205	3	12/01/2016	5	3	3	160	2	2	23/12/1943
208	3	04/02/2016	57	4	3			2	23/12/1943
855	2	17/05/2016	#REF!	5	3	219	2	2	01/05/1947
862	2	01/06/2016	#REF!	5	3			2	01/05/1947
576	2	25/08/2015	2	5	3	220	2	2	05/02/1932
629	2	01/09/2015	57	5	3			2	05/02/1932
311	3	12/02/2016	2521	4	3	161	2	2	06/10/1936
491	3	08/03/2016	696	5	3			2	06/10/1936
231	3	22/09/2015	1	4	3	162	3	2	19/01/1949
307	3	19/01/2016	206	4	3			2	19/01/1949
377	3	03/03/2016	682	5	3			2	29/08/1952
265	3	05/02/2016	2240	4	3	163	3	2	08/01/1952
287	3	01/02/2016	1957	4	3			2	08/01/1952
410	3	02/03/2016	898	5	3			2	08/01/1952
28	3	10/11/2015	674	1	3	164	3	2	12/11/1937
340	3	01/02/2016	2484	4	3			2	12/11/1937
497	3	10/03/2016	1260	5	3			2	12/11/1937
209	3	20/01/2016	736	4	3	165	3	2	07/12/1949
431	3	12/03/2016	123	5	3			2	07/12/1949
537	3	22/02/2016	3667	5	3			2	07/12/1949
822	2	13/06/2016	97	5	3	221	2	2	09/09/1953
873	2	20/06/2016	98	5	3			2	09/09/1953
591	2	26/08/2015	16	5	3	222	2	2	02/02/1953
814	2	13/06/2016	89	5	2			2	02/02/1953
376	3	27/02/2016	1102	5	2	166	2	2	28/09/1953
394	3	09/03/2016	1102	5	3			2	28/09/1953
32	2	11/11/2015	4	1	3	223	2	2	15/03/1942

778	2	09/06/2016	52	5	3			2	13/07/1939
1269	2	02/07/2015	35	2	3		2	2	
1930	2	07/06/2016	26	5	3			2	15-jun-28
780	1	20/03/2015	159	4	3	39	2	2	15/09/1939
820	1	24/03/2015	248	4	3			2	15/09/1939
207	2	22/02/2016	26	2	3	224	2	2	01/08/1928
258	2	06/08/2015	5	3	3			2	01/08/1928
39	3	19/11/2015	1622	2	3	167	2	2	15/08/2028
81	3	06/11/2015	568	2	3			2	15/08/2028
20	2	18/05/2015	18	1	2	225	2	2	03/04/1947
85	2	25/06/2015	28	2	3			2	
83	3	06/11/2015	439	2	3	168	2	2	11/07/1949
302	3	04/02/2016	190	4	3			2	11/07/1949
498	2	24/08/2015	94	4	3	226	2	2	01/01/1941
454	2	19/08/2015	49	4	3			2	01/01/1941
309	3	19/01/2016	123	4	3	169	2	2	02/08/1944
486	3	11/02/2016	222	5	3			2	02/08/1944
603	2	28/08/2015	28	5	3	227	2	2	13/11/1949
724	2	23/10/2015	175	5	1			2	13/11/1949
44	3	07/12/2015	1855	2	3	170	4	2	28/06/1942
78	3	13/11/2015	1904	2	3			2	28/06/1942
135	3	17/12/2015	2062	3	3			2	28/06/1942
349	3	25/01/2016	2860	4	3			2	28/06/1942
8	2	29/04/2015	7	1	3	228	2	2	14/03/1930
9	2	09/12/2015	34	1	3			2	14/03/1930
565	1	05/02/2015	397	3	3		2	2	18-jun-42
2457	3	24/02/2016	868	5	3			2	20-ago-43
702	1	27/02/2015	1760	2	3	40	3	2	29/05/1943
778	1	20/03/2015	154	4	3			2	29/05/1943
1143	1	06/05/2015	24240	5	3			2	29/05/1943
230	1	14/01/2015	235	2	3	41	2	2	04/11/1940
839	1	24/03/2015	211	4	3			2	04/11/1940
169	3	11/01/2016	1394	3	3	171	2	2	07/07/1938
313	3	01/02/2016	399	4	3			2	07/07/1938
236	3	15/09/2015	2861	4	1	172	2	2	21/01/1939
320	3	03/08/2015	286	4	1			2	21/01/1939
68	3	02/12/2015	1844	2	3	173	2	2	03/01/1939
140	3	17/12/2015	1288	3	3			2	03/01/1939
1718	2	17/06/2016	136	4	3		2	2	21-jan-34
1742	2	17/05/2016	162	4	3			2	incerta
573	2	23/05/2016	170	4	3	229	3	2	10/04/1928
641	2	03/09/2015	84	5	3			2	10/04/1928
155	2	14/07/2015	98	2	1			2	10/04/1928
276	1	15/01/2015	259	2	3	42	2	2	13/12/1934
1107	1	06/05/2015	257	5	3			2	11/08/1942
592	2	26/08/2015	17	5	2	230	2	2	25/03/1952
441	2	18/08/2015	36	4	3			2	25/03/1952
149	2	02/07/2015	92	2	3	231	2	2	02/06/1946
313	2	10/08/2015	60	3	3			2	02/06/1946
2123	3	27/11/2015	167	2	3		2	2	5-nov-49

2230	3	04/01/2016	329	3	3			2	5-nov-49
435	1	26/01/2015	169	3	3	43	3	2	29/04/1944
528	1	04/02/2015	416	3	3			2	29/04/1944
615	1	19/02/2015	416	3	2			2	29/04/1944
679	1	25/02/2015	376	1	3	44	2	2	21/02/1929
683	1	25/02/2015	73	1	3			2	21/02/1929
609	1	12/02/2015	355	3	3	45	3	2	29/01/1938
1047	1	29/04/2015	449	4	3			2	29/01/1938
663	1	11/02/2015	355	3	3			2	29/01/1938
444	1	27/01/2015	573	3	1	46	2	2	15/08/1936
765	1	18/03/2015	107	4	1			2	15/08/1936
605	1	12/02/2015	6214	3	1	104	2	1	25/01/1951
665	1	11/02/2015	6214	3	1			1	25/01/1951
446	1	27/01/2015	529	3	3	47	4	2	02/12/1937
458	1	27/01/2015	477	3	3			2	02/12/1937
569	1	05/02/2015	433	3	3			2	02/12/1937
681	1	25/02/2015	477	3	3			2	02/12/1937
237	3	11/09/2015	605	4	1	174	2	2	08/08/1940
322	3	05/08/2015	800	4	1			2	08/08/1940
428	1	26/01/2015	136	3	3	48	3	2	03/06/1936
735	1	16/03/2015	1100	4	3			2	03/06/1936
797	1	17/03/2015	90	4	3			2	03/06/1936
46	3	17/11/2015	1623	2	3	175	2	1	29/12/1936
253	3	20/01/2016	653	4	3			2	29/12/1936
943	1	15/04/2015	378	4	3	49	2	2	24/12/1941
479	1	28/01/2015	588	3	2			2	24/12/1941
603	1	12/02/2015	250	2	3	50	2	2	30/11/1937
260	1	15/01/2015	250	2	3			2	30/11/1937
948	1	15/04/2015	457	4	3	51	2	2	30/03/1948
1179	1	25/05/2015	356	5	3			2	30/03/1948
485	1	28/01/2015	553	3	3	52	2	2	07/02/1948
513	1	30/01/2015	977	3	3			2	07/02/1948
657	2	09/09/2015	102	5	1	232	2	2	10/10/1932
670	2	24/09/2015	115	5	3			2	10/10/1932
457	1	27/01/2015	494	3	3	105	2	1	25/07/1934
876	1	26/03/2015	31	5	3			1	25/07/1934
181	1	13/01/2015	112	2	2	53	2	2	03/08/1949
275	1	15/01/2015	307	2	3			2	
1151	1	04/05/2015	211	5	3	106	2	1	10/11/1936
1152	1	04/05/2015	1100	5	3			1	10/11/1936
233	1	14/01/2015	231	2	3	107	2	1	22/08/1941
471	1	27/01/2015	514	3	3			1	22/08/1941
602	2	28/08/2015	27	5	3	233	2	1	18/07/1945
803	2	13/06/2016	78	5	3			1	18/07/1945
101	1	15/12/2014	138	1	3	108	2	1	22/06/1940
725	1	11/03/2015	19	4	3			1	22/06/1940
46	1	10/12/2014	23	1	3	109	3	1	17/09/1929
134	1	10/12/2014	16	1	3			1	17/09/1929
690	1	26/02/2015	53	1	3			1	11/09/2029
563	1	05/02/2015	470	3	3	54	2	2	06/07/1929

913	1	13/04/2015	280	4	3			2	06/07/1929
868	2	15/06/2016		5	3	234	2	2	26/12/1957
793	2	07/06/2016	68	5	1			2	26/12/1957
636	1	23/02/2015	466	3	3	55	2	2	
184	1	13/01/2015	103	2	3			2	25/10/1925
612	1	12/02/2015	348	3	3	56	3	2	05/07/1939
661	1	11/02/2015	348	3	3			2	05/07/1939
680	1	25/02/2015	348	3	3			2	05/07/1939
652	1	11/02/2015	3102	3	3	110	3	1	06/04/1951
893	1	31/03/2015	76	5	3			1	06/04/1951
1070	1	27/04/2015	494	4	3			1	06/04/1951
148	1	12/01/2015	7266	2	3	111	2	1	24/02/1923
238	1	14/01/2015	215	2	3			1	24/02/1923
249	1	14/01/2015	182	2	3	112	2	1	15/08/1935
659	1	11/02/2015	199	3	3			1	15/08/1935
36	3	14/12/2015	498	2	3	176	3	2	12/04/1938
143	3	15/12/2015	309	3	3			2	12/04/1938
291	3	29/01/2016	1993	4	3			2	12/04/1938
705	2	16/10/2015	153	5	3	235	3	1	25/10/1945
728	2	09/11/2015	179	5	3			1	25/10/1945
729	2	09/11/2015	180	5	3			1	25/10/1945
121	3	07/08/2015	317	3	1	177	2	2	03/06/1938
144	3	05/08/2015	317	3	3			2	03/06/1938
358	2	11/03/2016	104	3	3	236	2	2	04/02/1929
560	2	17/05/2016	155	4	3			2	04/02/1929
111	3	15/09/2015	660	3	1	178	2	1	15/03/1942
154	3	29/07/2015	660	3	1			1	15/03/1942
190	3	22/12/2015	195	3	2	179	2	1	22/04/1947
239	3	19/08/2015	936	4	1			1	22/04/1947
233	3	18/09/2015	855	4	2	180	2	1	23/12/1935
333	3	08/09/2015	855	4	3			1	23/12/1935
27	1	10/12/2014	38	1	3	57	2	2	05/09/1945
141	1	10/12/2014	37	1	3			2	05/09/1945
79	1	15/12/2014	147	1	3	58	2	2	08/11/1939
637	1	23/02/2015	400	3	3			2	08/11/1939
677	1	25/02/2015	741	1	2	113	4	1	31/01/1931
689	1	25/02/2015	1555	1	2			1	31/01/1931
10	1	09/12/2014	1555	1	1			1	31/01/1931
12	1	09/12/2014	741	1	1			1	31/01/1931
13	3	08/10/2015	1889	1	3	181	2	2	10/03/1940
21	3	09/11/2015	1164	1	3			2	10/03/1940
496	1	29/01/2015	554	3	3	59	2	2	04/02/1950
874	1	26/03/2015	28	5	3			2	04/02/1950
480	1	28/01/2015	528	3	3	114	2	1	08/01/1930
962	1	22/04/2015	399	4	3			1	08/01/1930
894	1	31/03/2015	29160	5	3	115	2	1	20/05/1943
935	1	15/04/2015	99	5	3			1	20/05/1943
198	2	15/02/2016	15	2	3	237	3	2	16/08/1943
485	2	21/08/2015	81	4	3			2	16/08/1943
811	2	13/06/2016	86	5	3			2	16/08/1943

110	3	14/04/2015	156	3	3	182	2	2	24/11/1931
147	3	31/07/2015	206	3	3			2	24/11/1931
487	2	21/08/2015	83	4	2	238	3	2	11/11/1950
605	2	28/08/2015	30	5	3			2	11/11/1950
628	2	01/09/2015	56	5	3			2	11/11/1950
107	1	11/12/2014	61	1	3	60	2	2	03/02/1945
873	1	26/03/2015	27	5	3			2	03/02/1945
731	1	16/03/2015	83	4	3	61	2	2	05/03/1941
63	1	16/12/2014	149	1	3			2	05/03/1941
62	3	20/11/2015	219	2	3	183	4	2	28/02/1938
97	3	15/09/2015	92	3	3			2	28/02/1938
158	3	19/01/2016	1086	3	3			2	28/02/1938
336	3	04/01/2016	177	4	3			2	28/02/1938
91	3	27/11/2015	46	3	3	184	2	1	15/04/1937
213	3	19/01/2016	804	4	3			2	15/04/1937
614	1	19/02/2015	446	3	3	62	2	2	09/06/1943
1128	1	04/05/2015	446	5	3			2	09/06/1943
411	1	23/01/2015	2699	3	3	63	2	2	08/12/1940
621	1	20/02/2015	2699	3	3			2	08/12/1940
566	1	05/02/2015	399	3	3	64	2	2	31/07/1931
641	1	23/02/2015	399	3	3			2	31/07/1931
71	2	15/06/2015	14	2	3	239	3	2	
714	2	21/10/2015	163	5	3			2	02/05/1940
344	2	14/08/2015	91	3	3			2	02/05/1940
570	1	05/02/2015	381	3	3	116	4	1	21/06/1945
671	1	13/02/2015	516	3	3			1	21/06/1945
672	1	13/02/2015	51	1	3			1	21/06/1945
674	1	13/02/2015	7827	1	3			1	21/06/1945
3	2	29/04/2015	3	1	1	240	2	2	18/07/1948
830	2	15/06/2016	105	5	3			2	18/07/1948
806	1	17/03/2015	106	4	3	65	2	2	18/08/1943
916	1	13/04/2015	291	4	1			2	18/08/1943
73	3	13/11/2015	156	2	3	185	3	2	04/02/1941
74	3	20/11/2015	1983	2	3			2	04/02/1941
108	3	07/01/2016	1967	3	3			2	04/02/1941
16	1	09/12/2014	1592	1	3	117	3	1	29/06/1930
42	1	11/12/2014	60	1	3			1	29/06/1930
678	1	25/02/2015	60	1	3			1	29/06/1930
574	1	05/02/2015	396	3	1	118	2	1	19/05/1938
576	1	10/02/2015	210	3	3			1	19/05/1938
468	1	27/01/2015	501	3	3	119	2	1	19/11/1932
617	1	20/02/2015	501	3	3			1	19/11/1932
306	2	10/08/2015	53	3	1	241	2	1	08/03/1948
60	2	05/06/2015	3	2	1			1	08/03/1948
897	1	11/02/2015	225	2	3	120	3	1	21/06/1943
899	1	13/02/2015	4224	2	3			1	21/06/1943
1069	1	27/04/2015	493	4	3			1	21/06/1943
402	3	24/02/2016	825	5	3	186	2	2	08/07/1939
529	3	16/03/2016	1230	5	3			2	08/07/1939
184	3	02/12/2015	298	3	3	187	2	2	11/02/2027

413	3	12/02/2016	924	5	3	2	11/02/2027

IDAVIT	ESTCIVIT	PROVIT	INSTVIT	CORVIT	ACOMP	SEXOAGRE	DATNASAGRE	IDADEAGRE
63	3	1	2	1	2	1		
66	3	0	2	1	2	2		
66	3	0	2	1	2	1	19/02/1977	35
66	4	1	2	1	2	1		
66	4	1	2	1	2	1		
61	3	0		1	2	2	04/01/1965	46
62	3	0	8	1	2	1		
82	3	0	8	3	2			
82	3	0	8	3	2			
66	3	0	1	3	2	1	02/02/1973	38
67	3	0	1	3	2	1	02/02/1973	39
75	2	0	6	1	2	1		56
75	2	0	6	1	2	1		56
69	4	0	8	3	2	1	29/04/1972	39
71	3	1	8	3	2	1	29/04/1972	41
68	2	1	2	1	2	2	16/04/1972	39
69	2	0	2	1	2			
68	2	0	1	1	2	1	30/06/1974	36
68	2	0	1	1	2	1	30/06/1974	36
68	3	0	2	1	1	1	25/09/1965	46
70	3	0	2	1	1	1	25/09/1965	47
85	4		2		2	1	23/03/1974	37
85	3	0			1	1	17/06/1963	47
64	3	0	2	1	2	1		
64	3	0	2	1	2	1	25/04/1980	26
74	1	0	2	1	2	1		
76	3	0		1	1	1	09/02/1977	35
87	3	0	1	1	1	1	14/11/1988	24
87	3	0	1	1	2	1	14/11/1988	24
67	2		2	1	2	1		
70	2	0	4	1	2	1	23/06/1977	34
66	1	1	4	3	2			
67	1	1			2			
74	3	0	1		2	1	10/07/1968	44
74	3	0	1		2	1	10/07/1968	44
71	1	0	6	2	2	2	01/03/1965	47
71	1	0	6	2	2	1	11/12/1986	28
66	2	0	8	3	2	2		
66	2	0	8	3	2	2		
70	4	0	4	1	2	2		
73	2	0	2	1	2	1		74
67	4	0	4	1	2	2	11/05/1966	44
68	4	1	4	1	2	2		
62	2	0	2	1	2	1	09/11/1987	23
63	2	0	1	1	2	1		
65	5	0	2	1	1	2	21/07/1978	33
66	5	0	2	1	1	2	21/07/1978	33
93	2	0	3	3	2	1		
93					2	1		

93	2	0			2	1		
67	2	0			2	1	02/11/1972	38
67	2	0	1		2	1	02/11/1972	37
67	2	0	1		2	1	02/11/1972	37
67	2	0	4	3	2	1		
67	2	1	4	3	2	1		
75	1	0	2	1	2	2	28/06/1980	30
74	1	0	2	1	2	2		
69	5	0		1	2	1	08/08/1989	20
70	5	0		1	2	1	15/12/1949	60
80	3	0	2		2	1	19/11/1982	30
79	3	0	2	1	2	1	19/11/1982	30
81	3	0		1	1	1	19/11/1982	31
68	4 0		6		2	2	15/09/1988	
70	4				2	1	08/06/1978	35
69	5	0	2	1	2	2		
71	1	0	2	1	2	1		
81	2	0	4	1	2	1	20/10/1967	41
82	2	0	4	1	2	2	28/09/1958	51
83	2	0	1		2	2	05/06/1978	34
85	2	0	4		2	2	05/06/1978	37
70	3	0	2	1	2	1	17/03/1978	32
71	3	0	2	1	2	1		
75	3		2	1	1	2	02/03/1965	43
75	3		2	1	1	2	02/03/1965	43
75	3		2	1	1	2	02/03/1965	43
69	4		2	1	2	2	25/08/1992	18
70	4	0	2	1	1	2	25/08/1992	19
70	4	0	2	1	1	2	25/08/1992	19
70	4	0	2	1	2	2	25/08/1992	19
64	2	1	1	1	1	1	01/11/1992	19
62	2	1	1	1	2	1	01/11/1992	16
63	2	1	1	1	1	1	01/11/1992	19
64	2	1	1	1	1	1	01/11/1992	19
60	1	0	2	1	2	1	15/05/1987	23
60	1	0	4	1	2	1	15/05/1987	24
81	3	1	2	4	2	1	28/07/1963	46
83	3	1	2	4	2	1	28/07/1963	46
68	1	0	4		2	1	28/12/1972	63
70	4				2	1	16/10/1974	41
70	1		4		2	1	28/06/1971	41
64	1	0	2	3	1	2		
65	1	1		1	2	2		
60	4	1	2	1	2	1	06/01/1975	38
60	4	1	2	1	2	1	06/01/1975	38
61	2	1	6	1	2	1		34
62	2	1	6	1	2	1	23/10/1975	35
62	2	1	6	1	2	1	23/10/1975	35
62	2	1	6	1	2	1	23/10/1975	35
77	3	0		1	2	1	21/03/1955	56

77	3	0	8	1	1	1	21/03/1955	57
76	3	0		1	1	1	21/03/1955	55
69	4	1	4		2	1	02/08/1964	50
69	4	1	4		2	1	02/06/1964	50
84	4	0	2	1	2	2	14/01/1968	44
84	4	0	2	1	2	2	14/01/1968	41
69	3	0	1	1	2	1		
70	3	0	1	1	2	1		
63	2	1	4	1	2	2	30/12/1985	25
65	2	1	4	1	2	1	19/06/1951	60
64	1	1	2	1	2	1		
66	1	0	2	1	2	1		
71	3	0		1	1	1	17/03/1954	57
73	3	0	4	1	2	1		
75	3	0	2	1	2	1		
74	2	0	2	1	2	1		
75	3	0	2	1	2	2	13/09/1965	46
76	3	1	1	1	2	1		
69	2	1		1	2	1	12/10/1978	32
69	2	1		1	2	1	12/10/1978	32
76	1	0	4	1	2	1		
77	1	0	4	1	2	1		
72	4	0	2	1	2	1	04/11/1968	43
73	4	0	2	1	2	1	06/02/1973	39
66			6		2	1	24/10/1986	25
66	2	0	6		2	1	20/09/1956	56
66	4	0	6	1	2	2		
68	4	1	6	1	2	2		
88				1	1	2		
88				1	1	2		
69	2	0		1	1			
71	2	0	3	1	1	2	05/10/1955	56
80	3	1	2	1	1	2		
80	3	1	2	1	1	2		
73	3	0	2	3	2	2	25/03/1972	40
73	3	0	2	3	2	2	25/03/1972	40
63	3	0	1	3	1	1	04/11/1990	20
63	3	0	1	3	1	1	04/11/1990	20
72	2	0	2	1	1	1	08/08/1967	41
72	2		1	1	2	1	08/08/1967	41
60	3	0	2	3	2			
60	3	0	2	3	2			
69	3	0	1		2	1	17/12/1978	34
70	3	0	1		2	1	17/12/1978	35
62		1	1		2	1	04/03/1994	18
62		1	1		2	1	04/03/1994	19
69	2	0	1	3	2	2	18/02/1979	33
69	2	0	1	3	2	2	18/02/1979	33
63	3	1	2		2	2	13/09/1992	21
63	3	1	2	1	2	2	13/09/1992	21

80	3	0	1	3	2	1	12/01/1963	47
80	3	0	1	3	2	1	12/01/1963	47
		0	1		1	1	22/03/1979	32
64		0	1	3	2	1	22/03/1979	32
65	2	0	1	3	2	1	23/03/1979	33
65	2	0	1	3	2	1	23/03/1979	33
46	1	1	1	3	2	1	28/06/1984	27
60	1	0	2	3	2			
75	4	0	1		2	1	18/09/1984	29
75	4	0	1		2	1	10/09/1987	26
64	2	1	1		2	1	11/09/1989	21
65	2		3		2	1	06/05/1954	57
60	2	1	6	1	2	1	05/11/1988	21
60	2	1	6	1	2	1	05/11/1988	21
67	3	0	8		2	2	02/09/1968	44
67	3	0	1		2	1	16/02/1967	46
65	2	1	2	1	2	1		
65	2	1	2	1	1	1		
72	2	0	2	1	1	1		
72	2	0	2	1	2	1		
72	2	0	2		2	2	25/12/1990	19
61	4	0	2		2	2	13/10/1942	69
61	2	0	1		2	2	31/10/1942	71
68	2	0			1	2	23/07/1964	45
69	2	0	4		2	2	23/07/1964	47
65	2	0	1	1	2	1	29/04/1970	40
71	2	0	1	1	2	1	29/04/1970	42
69	3	0	4		2	1	22/11/1982	27
71	3	1	4		2	1	04/07/1979	32
63	6	1			2	1		
60	1	0	6		2	2	03/02/1987	22
60	1	0	6		2	1	18/07/1960	49
83	2				2			
82	1	0	8		2	2	11/12/1966	44
77	3	0	8		2	1	03/11/1975	37
80	3	0	8	3	2	1		
66	2	0			1	1		43
81	3	0	8		2	1		
80	3		8		1	1	incerta	40
74		0			2	1	23/02/1962	50
78	3	0	1		2	1		
74	2	1	1	3	2	1		
74	2	1	2	3	2	1		
76	3	1	1		2	1	28/12/1969	41
78	3	0	1		2	1	28/12/1968	53
68	3	0	2	3	1	2		
68	3	0	2	3	1			
63	2	0	2	1	2	1	05/04/1965	44
65	2	0	2	1	2	1	05/04/1965	47
67	1	1	2	1	2	2		

68		1	2	1	2	2	15/09/1990	20
68	5	1	2	1	2	2		
69	1	1	2	1	2	2		
76	1	0	8	2	2	2		
77	4	1	2	2	2	1		
77	4	0	2	2	2	2	26/02/1939	73
77	4	0	2	2	2	1	11/07/1958	55
77	4	0	2	2	2	2	26/02/1939	73
78	2	1	8	2	2	1	11/07/1958	55
83	5	0	1		2	2	24/12/1941	71
84	5	0	2	1	2	2		
72	2	0	2	2	2	1		
72	2	0	2	2	2	1		
60	2		2	1	2	1		
61	2	1	2	1	2	1		
70	2	0	2		2	2	10/03/1977	36
69	2	0	1		2	2	10/03/1977	36
73	3	0			2	2	17/05/1991	22
73	3	0			2	2	17/05/1991	22
71	4	0	1	1	2	1	20/09/1971	40
71	4	0	1	1	2	2	08/07/1973	39
84	2	0	2	1	2	2		
86	3	0	2	1	2	1	28/04/1977	34
70	2	0	2	3	2	1	26/06/1980	32
72	2	0	2	3	2	1	26/06/1980	33
71	2		3		2	1	20/12/1979	34
71	2		3		2	1	20/12/1979	34
63	3	0	8	3	2	2		27
65	3	0	8	3	2			
60	2	1	2	3	1	1	24/12/1963	45
62	2	1	2	3	2	1	01/02/1962	49
60	2	1	2	3	1	1	01/02/1962	46
80	2	0	1	3	2	1	19/11/1973	39
78	2		1	3	2	1	06/03/1983	30
63	2	0			2			
76	2	1	8	3	2			
61	2	1	6	1	2	1		
62	2	1	6	1	2	1	27/12/1964	48
67	2	0	6		2	2	12/11/1967	44
68	4				2	2	18/04/1960	52
71	2	0	2	2	2	1	01/12/1991	20
72	2		2	3	2	1		
67	2	1	4	1	2	1		15
69		1	4	1	2	2	28/09/1974	38
72	1	0	1		2	1	05/08/1959	51
72	1	0	1		2	2	02/01/1970	41
77		0			1	1	06/11/1974	36
78	2	0			2	1	06/11/1974	36
62	3				2	2	06/07/1989	24
61	4	1	1		2	2		45

60	2	1	6		2	1	10/10/1980	30
61	4	1	6		2	1	10/10/1980	32
84	3	0	8		2	2	16/01/1955	54
75	3	0	8		2	1		
72		0	8	3	2	1	04/03/1977	34
74	2	0	8	3	2	1	04/03/1977	36
66	2	1	4	1	2	1	03/06/1970	40
68	3		6	1	2	2	12/12/1973	38
73	2	1	2	3	2	1		
72	5	1	2	1	2	1	26/06/1981	28
70	2	0		3	2	1		
70	2	0		1	2	1	10/04/1974	36
70	2	0		1	2	1	10/04/1974	36
70	2	1	1		2	2	18/06/1987	25
70	2	1	2		2	1		
84	2	0	2		2	1		
85	2	0	2	1	2	1	21/02/1985	27
69	3	0	2	1	2	1	01/06/1961	48
69	3	0	2	1	2	1	01/06/1961	48
69		0		1	2	1	15/10/1993	16
71	5	0		1	1	1	15/10/1993	18
72	4	0	6	1	1			
73	4	0	6	1	2	1		
60	4	0	2		2	2	19/12/1965	43
72	1	0	2		2	1	07/06/1976	37
61	4	0	1		1	2	10/08/1960	50
77	4	0	8	3	2	2		
77	4	0	8	3	2	1		
61	2	1	1	3	2	1	10/08/1989	22
62	2		1	3	2	1	10/08/1989	24
61	2	0	1	3	2	2	07/07/1982	29
63	2	0	1		2	2	07/07/1982	30
63	2	1	1	1	2	1	21/05/1970	40
65	2	0	1	1	2	1		
71				1	2	1		
74	3	0	1	1	2	1		
74	3	0	1	1	2	1		
66	3	0	8		2	1		42
62		1			2	1	06/09/1971	39
73	4	0	1	1	2	2		
73	4	0	1	1	1	2		
93	4	0	4	3	2	2	17/10/1960	51
94	4	0	4	3	2	2	17/10/1960	53
94	2	0	4	3	2			
68	3	0	8	3	2	1	02/02/1985	26
68	3	0	8	3	2	1	02/02/1985	26
66	4	0	6	1	2	1	26/02/1980	32
66	4	0	1	1	2	2		
65	3	1	2	3	2	1	09/02/1980	32
66	3	0	2	3	2	1	09/02/1980	33

70	3	1	1		2	2	29/07/1971	40
70	3	1	1		2			
65	4	0	8	3	2	2		
67	3	0	8	1	2	2		
65	3	1	2	1	2	1		36
62	1		2	1	2	1	08/04/1979	36
68	3	0	2	1	1	2	14/03/1963	48
68	3	0	2	1	1	2	14/03/1963	48
69	1	0	4		2	2	09/04/1972	39
70	1	0	4		2	1	11/09/1946	66
73	1		1		2	1	05/09/1978	33
72	3	1	1		2	1	05/09/1979	33
66	2	1	2	3	2	1	13/04/1987	24
67	2	1	2	3	2	1	13/04/1987	25
80	3	0	1	1	2	1	22/01/1954	57
82	3	0	1	1	2	1	24/07/1960	52
61	2	1	8	2	2	1	07/10/1975	35
63	2	0	8	2	2	1	07/10/1975	37
74	4	1	2		2	2	07/08/1975	57
73	4	1	1		2	2	23/06/1988	23
65	1			1	2	2	25/11/1947	64
65	1	0	6	1	2	2		
65	1	0	6	1	1	2	28/09/1958	54
68	4	0	1	2	2	1	18/06/1943	68
68	4	0	1	2	2	1	18/06/1943	68
69	3	1			2	2		33
69	3	1		3	2	2	26/01/1983	33
81	3	0	8		2	2	19/10/1977	36
81	3	1	8		2	2	19/10/1977	36
75	3	1	2		2	1	15/11/1987	24
77	3	1	2		2	1	15/11/1987	26
63	3	0	8	3	2	1	16/08/1989	23
63	3	0	8	3	2	1	16/08/1989	23
61	2	0	2		2	1	16/08/1980	33
60		1	4	3	2	1	22/02/1984	28
60	2	1	4	3	2	1	22/02/1984	28
61	2	1	4	3	2	1	22/02/1984	29
71	3	0	2	1	2	1	25/03/1983	25
71	3	0	2	1	2	1	25/03/1983	26
76	3	0	2	1	2	1	25/03/1983	30
62	2	1	1	3	2	1	24/09/1989	22
64	2				2	1	24/04/1989	24
63	2	1	1		2	1	24/04/1989	24
60	2	1	6		2	1	15/10/1946	67
62	2	1	6	3	2	1	15/10/1946	68
62	4	1	4		2	2	18/02/1974	39
60	4	1	4	3	1	1	20/02/1978	36
60	2	0	3		2	1	20/01/1984	29
60	2	0	3	3	2	1	20/01/1984	29
67	2	1	2		2	1	26/03/1959	50

70				2	2	2	08/07/1968	45
81	3	Aposentada	6		2	1	4-mar-58	52
82	3	Pensionista	6		2	1		
72	3	0	2	1	2	1		
72	3		2	3	2	1	14/06/1960	51
81	2	0	1		2	1	31/01/1981	29
82	2	0	2		2	1	31/01/1981	30
81	3	0	1	3	2	1	16/05/1950	60
81	3	0	1	3	2	1	16/05/1950	60
62	4	0	6		2	2	09/07/1949	60
62	4	0	6		2	1	04/03/1970	40
61	1		8	2	2	1	04/04/1975	35
63	1	0	8	2	2	1	04/04/1975	37
71	2	0	1		2	2	18/02/1958	54
71	2	0	4		2	2	18/02/1958	54
68		0	8		2	1	19/01/1981	31
68	2	0	8		2	1	19/01/1981	32
62	1	0	1		2	2	14/03/1959	54
63	1	0	2		2	2	14/03/1959	54
68	3	0	3	3	2	1	01/04/1972	38
68	3	0	8	3	2	1	01/04/1972	38
68	3	1	8	3	2	1	01/04/1972	39
69	3	0	8	3	2	1	01/04/1972	40
79	3	0	1		2	2	16/11/1974	34
79	3	1	2		2	2		
69	3	Aposentado		1	1	1		
70	2	Doméstica	1	3	2	1	8-abr-84	29
66	2	1	2	1	2	2	15/02/1978	31
68	3	0	2	1	2	1		
70	3	0	2	1	1	1		
70	4	0	1	1	2	1	31/12/1964	45
71	4	0	1	1	1	1	31/12/1964	47
72			1		2	1	26/12/1974	37
73	1	0		3	2	1	26/12/1974	37
73	2	1	6	3	2	1		
73	2	0	6	3	2	1		
71	4	0	4	3	2	1	20/08/1972	38
72	4	0	4	1	2	1	20/08/1972	38
78	1				2	2		
77	1	Do lar	1		1	2	30-set-88	24
84	3	1			2	2	10/12/1964	47
85	3	1	4		2	1	28/02/1967	46
82	3	1	4		2	1	20/09/1956	54
75	1	0	2	1	2	1		
70	5	0	1	2	2	1		
61	2	1			2	1	14/08/1977	36
60		1	3		2	1	14/08/1977	34
64	3	1	8		2	1	01/05/1965	45
65	3	0	8		2	2	05/09/1969	41
61	2	Doméstica	1	3	2	1	9-nov-73	37

61	2	Doméstica	1	3	2	1	9-nov-73	38
66		0	6	1	2	2	23/06/1981	29
67	1	0	6	1	2	1		
67	1	0	6	1	2	1		
80	3	0	1	1	1	1	27/09/1949	60
80	3	0		1	1	2	17/06/1955	54
73	4	0	2	1	1	1	10/10/1973	37
74	4		2	1	2	2	20/06/1960	52
73	4	0	2	1	1	1	10/10/1973	37
75	3	0	2	1	2	2	30/01/1948	64
75	3	0	2	1	2	2	30/01/1948	64
60	4	1	1	1	2	1		
60	4	1	1	1	2	1		
73	3		2	1	2	2		37
73	3	1	2	1	2	1	11/08/1963	48
73	3	1	2	1	2	1	11/08/1963	48
73	3	1	2	1	2	1	11/08/1963	48
72	3	0	4	2	2	1		
72	3	0			2	1		
74	2	1		1	2	1	06/01/1960	51
75	3	1	2	1	2	2		
75	3	0		1	2	1	12/02/1977	35
73		0	1		2	1	07/03/1963	47
75	2	0	1	3	2	1	07/03/1963	49
70	1	0	2	1	2	1		
69	1			1	1	1	05/03/1959	52
72	3		2	1	2	1		
72	3		2	1	2	1		
64	1	0	1	1	2	1		
65	1	0	2	1	2	1		
63	4	0	2	1	2	2	18/12/1970	40
63	4	0	2	1	2	2	18/12/1970	40
80	1	1	8		2	1	03/02/1995	18
81	1	1	8		2	1	03/02/1995	18
77	2	0	2	3	1	2		
78	2	0	2	1	2	2	08/03/1964	49
60		0	2	1	2			
60	2	0	2	1	1	1	20/08/1966	44
76	3	0	1	1	2	1		
76	3	0	1	1	2	1		
69	2	0	2	1	2	2	26/12/1970	39
70	2	0	2	1	2	2	27/02/1941	70
67	3	0	6		2	2	11/10/1948	65
69	3	0	6		2	2		
69	2	1	4	1	2	1	16/04/1975	34
71	2	0	4	1	2	1	16/04/1975	36
79	2	0	2	1	2	1		
79	2	0	2	1	1	1		
79		0	2	1	2	1	27/09/1979	30
82	3	0	1	1	2	1		

82				1	2	1	17/11/1964	47
59	1	0	1		2	1	12/05/1969	47
68	1	0	4		2			
85	3	0	8	1	1	2		
84					2	1		
72	3	0	1	1	1	2	25/02/1971	40
72	3	0	1	1	1	2	25/02/1971	40
72	3	0	1	1	1	2	25/02/1971	40
60	2	0	2	3	1	1		24
61	4	0	2		2	2	27/09/1962	50
61	1	1	2	3	2	1	16/01/1987	26
87	4	0	2	1	2	2	13/06/1988	22
87	4	0	2	1	2	2	13/06/1988	22
75	2	0	2	1	2	1	22/04/1964	46
75	2	0	2	1	2	1	22/04/1964	47
72		0	1		2	1	08/09/1970	40
73	2	0	1		2	1	28/07/1964	47
74	2	0	1	3	2	1	28/07/1964	48
68	2	1	1		2	1	14/12/1979	34
68	3	1	1		2	1	18/04/1980	33
68	2	1	1		2	1	28/08/1971	42
73	3	0	2	3	2	1		
73	3	0	2	3	2	1		
82	3	0	6	1	2	1	22/04/1958	52
83	3	0	1		2	2		
69	1	0	1	3	2	2		
69	1	0	1	3	2	2		
64	4	1	4		2	1	27/05/1978	33
65	4	0	4	3	2	1	04/05/1976	36
76	2	1	6	3	2	1	19/10/1986	26
76	2				2	1	19/10/1986	26
63	1		6	1	2	1	22/10/1969	39
63	1		6	1	2	1	22/10/1969	39
70	1	0		1	2	1	15/02/1974	35
71	1	0	6	1	2	1	18/01/1982	28
78	2		2	2	1			
78	4	0	2	2	2	1		
78	4	0	2	3	2			
78	2		2	2	2			
69	2	0	8		1	1	26/03/1943	66
69	2	0	8		2	1	26/03/1943	66
61		1	1	1	2	1	06/07/1976	35
62	2	1	1	1	2	1		
81	3	0	1	1	1	1		
82	3	0	1	1	2	1		35
69			3		2	1	02/08/1960	52
69	1	0	3	1	2	1		
64	3	1	4		2	1		42
69	3	1	4		2	1	09/12/1981	30
72					1	1	07/10/1967	43

80	2	0	3	3	2	2		
80	2	0	3	3	2	2		
62	4	0	6		2	1	28/03/1964	48
62	4	0	6		2	1	28/03/1964	49
62	4	0	6		2	1	28/03/1964	49
64	4	0	1	3	1	1	18/08/1986	23
67	4	0	2	1	2	1	03/02/1964	48
70	3	0	1	1	1	1		
68	3	0		1	2	1	09/11/1964	45
72	1	0	8	3	2	1		
72	1	0	8	3	2	1	08/10/1975	36
73	1	0	8	3	2	1	08/10/1975	35
73	1	0	8	3	2	1	08/10/1975	36
73	3	0	8	3	1	1	04/01/1972	39
75	3	0	8	3	2	1		
70	3	0	2	1	1	1	30/03/1961	52
70	3	0	2	1	1	1	30/03/1961	52
70	3	1	2	1	2	2		
70	3	1	2	1	2	2		
80	3	0		1	1	2	06/10/1968	43
80	3	0	8	1	1	2	06/10/1968	43
69	2	0	8		2	1	20/10/1951	58
73	1		2		2	2	06/09/1965	48
71	1	0	1		2	2	06/09/1965	46
66	2	0	2	1	1	1	01/11/1992	18
66	2	0	1	1	1	1	01/11/1992	19
63	2	0	1	1	2	1	01/11/1992	16
64	2	0	1	1	1	1	01/11/1992	17
61	3	1	6		2	1	10/05/1975	34
65	3	0	6		2			
68	3	1		1	2	2		
68	3	0	4	1	2	2		
69	3	1	2	3	2	1	25/08/1977	33
69	3	1	2	3	2	1	25/08/1977	33
69	3	1	2	3	2	1	25/08/1977	33
78	2	0	8	2	2	1		42
79	2		8	3	1	1	17/07/1961	48
79	2		1	3	1	1	17/07/1961	48
73	2	0	4	1	2	2	06/05/1970	41
72	2	0	4	1	2	1	20/10/1958	52
78	4	0	2	1	2	1		
78	4	0	2	1	2	1		
63	2	0	6		2	2	05/02/1958	53
61	2	1	6		2	2	05/02/1958	51
67		1	2	1	2	1		
66	3	1	2	1	2	1		
69	3	1	2	1	2	1		
74	2	0	1	2	2	1	10/09/1994	19
74	2	0	1	2	1	1	10/09/1994	19
84	3	0	2	3	2	1	18/01/1976	35

86	3	0	2	3	2	1	18/01/1976	37

ESTCIVAGRE	INSTAGRE	**FAMILIAR**	**FAM**AGRE	**NFAMILIAR**	**NFAM**AGRE	CORAGRE	MORAIDOSO
1		1	2	2		1	1
		2		1	5	1	2
1	2	1	2	2		2	1
2		1	5	2		1	1
		1	5	2		1	1
2	2	2		1	2		2
		2		1	5	1	2
		2		1	5		0
		2		1	5		0
1	1	1	2	2		3	1
1	1	1	2	2		3	1
2	6	1	5	2		1	2
2	3	1	5	2		1	2
1	2	1	2	1		3	1
1	2	1	2	2		3	1
1		1	2	2		1	2
		1	3	2			1
2	1	1	2	2		1	1
2	1	1	2	2		1	1
1		1	2	2		1	1
1	2	1	2	2		1	1
5	3	2		1	5		2
5	1	2		1	2		2
		1	2	2		1	1
1	6	1	2	2		1	2
1		1	2	2		1	1
1		1	2	2		1	1
1		1	3	2		1	1
1	1	1	3	2		1	1
		2		1	5	1	2
2		2		1	2	1	2
		2		1	5		0
		2		1	5		0
1		1	2	2			1
1		1	2	2			1
2		1	6	2		3	2
1	3	1	4	2		2	1
	6	2		1	5	3	0
	6	2		1	5	3	0
5		1	2	2			1
		2		1	1	1	2
4	1	1	7	2		1	2
4		2		2	5	1	2
1	2	2		1	5	1	2
1		1	2	2		1	1
1		2		1	5	1	1
1	4	1	2	2		1	2
		2		1	5		0
		2		1	5		0

		2		1	5		0
1		1	2	2			1
1	1	1	2	2			0
1	1	1	2	2		3	0
		2		1	5		0
		2		1	5		0
		2		1	5	1	2
		2		1	5	3	2
		1	2	2		1	1
5		1	7	2		1	2
1	3	1	2	2		1	1
1	1	1	2	2			1
1	1	1	2	2		1	1
1	4	1	7	2			2
2	1	2		1	4		2
2		1	1	2		5	1
		1	2	2			1
1	2	2		1	5	2	2
		2		1	2	1	2
5	1	1	5	2			2
2	1	1	7	2		3	2
1	4	1	2	2		1	2
5		1	2	2		1	2
2	4	2		1	3	1	2
2	4	2		1	3	1	2
2	4	2		1	3	1	2
		1	2	2		1	1
1	2	1	3	2		1	1
1	2	1	3	2		1	1
1	2	1	3	2		1	1
1	2	1	3	2		1	1
1	2	1	3	2		1	1
1		1	3	2		1	1
1	2	1	3	2		1	1
1	4	1	7	2		1	1
4	4	1	7	2		1	2
5	1	1	2	2		4	0
5	1	1	2	2		4	1
1	2	2		1	2		2
2	4	2		1			2
2	4	2		1	5		2
		1	2	2		1	1
2		2		1	5	1	2
1	1	1	2	2		1	1
1	1	1	2	2		1	1
		1	2	2		1	2
5		1	2	2		1	2
5		1	2	2		1	2
5		1	2	2		1	2
1	2	1	2	2		1	1

1	2	1	2	2		1	1
1	2	1	2	2		1	1
1	1	2		1	2		2
4	1	2		1	2		2
4		2		1	5	3	2
1	2	2		1	5	3	2
		2		1	2	1	2
5		2		1	2	1	2
1		1	2	2		1	1
2		2		1	2	1	2
		2		1	5		2
		2		1	5	1	2
1		1	7	2		1	2
		2		1	5	3	2
1		1	3	2		1	1
1		1	3	2		1	2
		2		1	2		2
		2		1	2		2
4		1	2	2		1	1
4		1	2	2		1	1
		2		1	2		2
		2		1	5		1
2	4	1	2	2		1	2
4	2	2		1	2	1	2
	4	2		1	5		2
2	6	1	1	2			1
		2		1	4	3	2
		2		1	2	3	2
		1	2	1			
		1	2	2		1	1
3		2		1	2	1	2
		1	5	2			1
		1	5	2			1
2	2	2		1	2	3	0
2	2	2		1	2	3	0
1		1	2	2		3	1
1		1	2	2		3	1
1	4	2		2	1	1	1
1	4	2		1	5	1	1
		2		1	5		0
		2		1	5		0
1	1	1	2	2			1
1	1	1	2	2			1
1	1	1	2	2			1
1	1	1	2	2			1
1	1	1	2	2		3	1
1	1	1	2	2		3	1
1	1	1	4	2			1
1	1	1	4	2		1	1

2	1	1	2	2		3	0
2	1	1	2	2		3	0
1		1	3	2			1
1	1	1	3	2			1
1	1	1	2	2		3	1
5	1	1	2	2		3	1
5	3	1	5	2		2	1
		2		1	5		0
1		1	5	2			2
5		1	5	2			1
1	4	2		1	5		2
2	3	2		1	2		2
1	5	2		1	5	1	0
1	5	2		1	5	1	0
1	1	2		1	2		2
1	1	2		1	2	3	2
		2		1	5	1	2
		2		1	5	1	2
5		2		1	5	1	2
		2		1	2	1	2
5		2		1	2	1	2
4	2	1	1	2			2
4	2	1	1	2			1
2		1	1	2			1
2	2	1	1	2			1
1	1	1	2	2		1	1
1	2	1	2	2		1	1
1	4			2	5		2
2	6	2		1	2		2
		2		1	5		1
1	5	2		1	2		2
2	6	2		1	5		2
1	2	2		1	2		2
2	1	1	3	2			2
	1	2					2
1	4	1	2	2			1
		1	2	2			1
1		1	2	2			1
2	6	2		1	5		2
		1	2	2	não compareceu		1
		2		1	5	1	1
		2		1	5		2
2	4	2		1	2		2
2	4	2		1	5		2
2		2		1	2		2
		2		1	2		2
3	2	1	2	2		1	1
3	2	1	2	2		1	1
		1	1	2		3	1

5		1	7	2		3	1
5		1	7	2		3	1
		2		1	1	2	2
4		2		1	5	2	1
		1	2	2		2	2
4		2		1	5	2	1
1	2	1	2	2		2	2
4		2		1	5	2	1
1	2	1	2	2		2	1
5		1	1	2		1	2
		1	7	2		1	1
		2		1	5		0
		2		1	5		0
		2		1	5		2
		2		1	5		2
2	4	2		1	2		2
	5	2		1	2		2
1		1	3	2			1
1		1	2	2			1
2	2	2		1	2	3	2
2	2	2		1	2	1	2
		2		1	3		2
1	4	2		1	5	2	2
1	1	1	2	2		3	1
1	1	1	2	2		3	1
5	4	1	2	2		1	1
5	4	1	2	2		1	1
1	8	2		1	5	3	0
		2		1	5		0
1	2	1	2	2		1	2
1	6	1	7	2		1	2
1	6	1	2	2		1	1
1	1	1	3	2		3	1
1	1	1	2	2		3	1
		2		1	5		0
		2		1	5		0
4		1	4	2		1	2
4		1	4	2		3	2
4	2	1	1	2		1	1
1	6	2		1	4		2
5		1	2	2		2	1
1		1	2	2		3	1
1		2		1	2	1	2
2		2		1	2	1	2
2	3	2		1	5		2
2	4					1	2
1	2	1	2	2			1
1	2	1	2	2			1
1		1	7	2			1
2	3	1	6	2			1

1	6	2		1	2		2
2		2		1	2		2
2	2	2		1	2		2
							2
1	1	1	2	2		3	1
1	1	1	2	2		3	1
4	6	1	2	2		1	2
1		1	2	2		1	2
1	1	1	2	2		1	1
1		1	2	2		3	1
1		1	2	2		3	
1		1	2	2		1	1
1		1	2	2		1	1
2	5	2		1	2		2
1	6	2		1	2		2
1		1	2	2		3	1
1	2	1	2	2		3	1
4	2	2		1	5	1	2
4	2	2		1	5	1	2
1	2	1	2	2		3	1
1		1	7	2		2	1
		2		1	5		2
		2		1	5		2
2	6	2		1	2		2
4	4	2		1	2		2
1	6	2		1	2		2
4		1	1	2			1
		2		1	1		1
1	1	1	3	2		3	1
1	1	1	3	2		3	1
1	3	1	2	2		3	1
1	3	1	2	2			1
4		1	2	2		1	1
		1	2	2		1	1
2		2		1	2	1	2
		2		1	2	1	2
		2		1	2	1	2
1		1	2	2			1
1	1	1	2	2			2
		2		1	2	1	2
		2		1	2	1	2
1	8	1	2	2		3	1
1	8	1	2	2		3	1
			2	2	5		0
1	2	1	2	2		3	1
1	2	1	2	2		3	1
2		2		1	2	1	2
		2		1	2		2
1	2	1	2	2		3	1
1	2	1	2	2		3	1

4	1	1	2	2			0
		2		1	5		0
2		1	2	2		1	1
		1	2	2		1	1
4		1	2	2		1	1
		1	2	2		1	2
2		1	2	2		1	2
2		1	2	2		1	2
	4	1	4	2			2
2	1	1	6	2			2
1	1	1	2	2		3	1
1	1	1	2	2		3	1
1	1	1	3	2		3	1
1	1	1	3	2		3	1
2		2		1	2	1	2
4	2	1	2	2		1	1
1	1	1	2	2		2	1
1	1	1	2	2		2	1
1		2		1	1		2
4	1	2		1	5		2
2		2		1	5	1	2
		2		1	2	1	2
4		2		1	2	1	2
4	2	1	2	1	5	2	2
4	2	1	2	1	5	2	0
		1	2	2			1
1	1	1	2	2		3	2
5	1	2		1	2		2
1	1	2		1	2		2
1	2	1	2	2			1
1	2	1	2	2			1
1	1	1	2	2		3	1
1	1	1	2	2			1
2	2	1	2	2		3	1
1	1	1	2	2		3	1
2	1	1	2	2		3	1
2	1	1	2	2		3	1
1	1	1	3	2		1	0
1	1	1	3	2		1	0
1	1	1	3	2		1	0
1	1	1	2	2		3	1
1	1	1	2	2			1
5	1	1	2	2			1
2	3	1	1	2		3	1
2	4	1	1	2		3	1
5	4	1	5	2			2
2	4	1	2	2			1
1	2	1	2	2			1
1	2	1	2	2		3	0
2	1	1	2	1			2

1	2	1	2	2		3	2
2	4	2		1	2		0
		2		1	2		0
1		1	2	2		1	1
1		1	2	2		3	1
5		2		1	2	3	2
5	4	2		1	2		2
2	1	1	2	2		3	1
2	1	1	2	2		3	1
4	4	2		1	2		2
5	2	2		1	4		2
1	1	1	2	2		2	1
1	1	1	2	2		2	1
4	6	2		1	2		2
2	6	2		1	2		2
1		1	2	2			1
1	1	1	2	2			1
2		2		1	2		2
2		2		1	2		2
1	3	1	2	2			1
1	1	1	2	2		3	1
1	1	1	2	2		3	1
1	1	1	2	2		3	1
3	3			2	5		2
		2			1		2
		1	2	2		1	1
1	1	1	2	2		3	1
5	1	2		1	2	3	2
		2		1	5	1	2
							2
3		1	2	2		3	1
1		1	2	2		1	1
2	1	1	4	2			1
2	1	1	4	2			1
		2		1	5		0
		2		1	5		0
1	4	1	2	2		1	1
1	4	1	2	2		1	0
		1	3	2			1
1	1	1	3	2			1
5	2	2		1	4	3	2
2	4	2		1	4		2
2	6	2		1			2
4		1	5	2		2	2
5		1	1	2		3	1
2		2		1	2		2
2	4	2		1	1		2
1	4	2		1	2		2
2	4	2		1	2		2
1	1	1	2	2		3	1

1	1	1	2	2		3	1
5	2	2		1	2	1	2
		2		1	2	1	2
		2		1	2	1	2
2	1	1	2	2		1	2
3	2	1	3	2		1	1
1		1	2	2		3	1
1		2		1	2	1	2
1		1	2	2		3	1
1		2		1	5	1	2
1	4	2		1	5	1	2
		2		1	5		2
		2		1	5		2
5		2		1	5	1	2
4		1	2	2		1	1
4		1	2	2		1	1
4		1	2	2		1	1
2		2		1	5		0
		2		1	5		0
		2		1	2	1	2
		2		1	2	1	2
5		1	3	2		1	2
1	2	1	2	2			1
1	2	1	2	2		3	1
2		2		1	5	1	2
1		2		1	5	1	2
4		1	2	2		2	1
4		1	2	2		3	1
1		1	6	2		1	1
1	2	1	6	2		1	1
1	2	2		1	2	1	2
1		2		1	2	1	2
1	3	2		1	2		2
1		2		1	2		2
		1	2	2			1
1		1	2	2		3	1
1	2	2		1	5	1	2
		2		1	5	1	2
		2		1	5		2
		1	2	2		1	2
2	1	1	1	2		1	1
		2		1	2		2
		2		1	1		2
1	2	1	5	2		3	1
2	2	1	5	2		3	1
1		2		1	5	3	2
		2		1	5	3	2
		2		1	5	1	2
1		1	2	2		1	1

1		1	2	2		1	1
2	4	2	7	1	5		2
		2		1	5		2
	6	1	2	2		1	1
		2		1	5		2
2		1	2	2		1	2
2		1	2	2		1	2
2		1	2	2		1	2
		1	7	2			1
5		2		1	5	1	1
1		1	7	2		1	1
1	2	1	3	2		1	2
1	2	2		1	5	1	2
2	4	1	2	2		1	1
2		1	2	2		1	1
		1	2	2			1
4	1	1	2	2			1
4	1	1	2	2		3	1
1	6	2		1	5		2
2							2
1		2		1			2
4	1	1	3	2		3	0
4	1	1	3	2		3	0
1	2	1	2	2			2
		1	2	2			1
		2		1	5		0
		2		1	5		0
1	5	2		1	5		0
1	6	2		1	5	3	0
		2		1	5		0
		2		1	5		0
1	6	2		1	5	1	2
1	6	2		1	2	1	2
		2		1	5	3	2
1		2		1	2	1	1
		2		1	4		2
		2		1	4		2
							2
2		1	1	2			1
2	4	1	1	2			1
		1	4	2		1	2
5	1	1	4	2		1	1
		1	2	2		1	1
1		1	2	2		1	1
2		2		1	5	3	2
		1	2	2		1	1
		1	2	2			1
1	4	2		1	2		2
1	4	1	2	2			1

		2		1	1		0
		2		1	1		0
1	1	2		1	5		2
1	1	2		1	5		2
	1	2		1	5		2
1	1	1	3	2		3	1
1		1	2	2		1	1
3		1	2	2		1	1
3		1	2	2		1	1
1	1	1	2	2		3	1
1	1	1	2	2		3	1
1	1	1	2	2		3	1
1	1	1	2	2		3	1
1	1	1	2	2		3	1
1	8	1	2	2		3	1
4		1	2	2		1	2
4	6	1	2	2		1	1
2		2		1	2		2
		2		1	2	1	2
5		1	2	2		1	1
4		1	2	2		1	1
2	1	2		1	5		2
2	8	2		1	2		2
2	1	2		1	2		2
1	2	1	3	2		1	1
1	2	1	3	2		1	1
1	2	1	3	2		1	1
1	1	1	3	2		1	1
1	4			1	5		2
		1	6	2			
1		2		1	2	1	2
		2		1	2	1	2
1	3	1	2	2		3	1
1	3	1	2	2		3	1
1	3	1	2	2		3	1
		1	2	2		3	1
4	2	1	2	2		3	1
4	2	1	2	2		3	1
2		2		1	2	1	2
1	2	2		1	2	1	2
		2		1	2	1	2
		2		1	5	1	2
3	8	2		1	5		2
2	8	2		1	5		2
		2		1	5		2
		2		1	2	1	2
		2		1	5		2
1	1	1	3	2		2	1
1	1	1	3	2		3	1
1	1	1	3	2		3	1

1	1	1	3	2	3	1

VIOFIS	VIOPSIC	VIOSEX	VIOFINAN	VIONEGLI	VIOAUTO	VIOABAN	TOTVIO	ENCIML
1	1	0	0	0	0	0	2	1
0	0	0	1	0	0	0	1	0
1	1	0	0	0	0	0	2	1
0	1	0	0	0	0	0	1	0
0	1	0	0	0	0	0	1	0
0	1	0	0	0	0	0	1	0
0	1	0	0	0	0	0	1	0
0	0	0	1	0	0	0	1	0
0	0	0	1	0	0	0	1	0
0	1	0	1	0	0	0	2	0
0	1	0	0	0	0	0	1	0
0	1	0	0	0	0	0	1	0
0	1	0	0	0	0	0	1	0
0	1	0	1	0	0	0	2	0
0	1	0	1	0	0	0	2	0
1	1	0	0	0	0	0	2	0
1	1	0	1	0	0	0	3	0
1	1	0	0	0	0	0	2	1
1	1	0	0	0	0	0	2	1
0	1	0	1	0	0	0	2	0
0	1	0	0	0	0	0	1	0
0	0	0	1	0	0	0	1	0
0	1	0	0	0	0	0	1	0
1	1	0	0	0	0	0	2	1
1	0	0	0	0	0	0	1	1
0	1	0	1	0	0	0	2	0
0	1	0	1	0	0	0	2	0
0	0	0	1	0	0	0	1	0
0	1	0	1	0	0	0	2	0
1	1	0	0	0	0	0	2	0
0	1	0	0	0	0	0	1	0
0	0	0	1	0	0	0	1	0
0	0	0	1	0	0	0	1	0
0	1	0	0	0	0	0	1	0
0	1	0	0	0	0	0	1	0
0	1	0	0	0	0	0	1	0
0	1	0	0	0	0	0	1	0
0	0	0	1	0	0	0	1	0
0	1	0	1	0	0	0	2	0
1	1	0	0	0	0	0	2	0
1	0	0	0	0	0	0	1	0
1	0	0	0	0	0	0	1	1
0	1	0	0	0	0	0	1	0
0	1	0	0	0	0	0	1	0
0	1	0	0	0	0	0	1	0
0	1	0	0	0	0	0	1	0
0	1	0	0	0	0	0	1	0
0	0	0	1	0	0	0	1	0
0	1	0	1	0	0	0	2	0

0	1	0	1	0	0	0	2	0
0	1	0	1	0	0	0	2	0
0	1	0	1	0	0	0	2	0
0	1	0	1	0	0	0	2	0
0	0	0	1	0	0	0	1	0
0	0	0	1	0	0	0	1	0
0	1	0	0	0	0	0	1	0
1	0	0	1	0	0	0	2	1
1	1	0	0	0	0	0	2	1
0	1	0	0	0	0	0	1	0
1	1	0	0	0	0	0	2	1
1	0	0	0	0	0	0	1	0
0	1	0	1	0	0	0	2	0
0	1	0	0	0	0	0	1	0
0	1	0	0	0	0	0	1	0
1	0	0	0	1	0	0	2	0
1	1	0	0	0	0	0	2	0
0	1	0	0	0	0	0	1	0
0	1	0	0	0	0	0	1	0
0	1	0	0	0	0	0	1	0
1	0	0	1	0	0	0	2	1
1	0	0	0	0	0	0	1	0
1	1	0	0	0	0	0	2	0
0	0	0	0	1	0	0	1	0
0	0	0	0	1	0	0	1	0
0	0	0	0	1	0	0	1	0
0	1	0	0	0	0	0	1	0
0	1	0	0	0	0	0	1	0
1	1	0	0	0	0	0	2	0
1	1	0	0	0	0	0	2	1
1	1	0	1	0	0	0	3	1
1	1	0	1	0	0	0	3	1
1	1	0	0	0	0	0	2	0
1	1	0	0	0	0	0	2	1
0	0	0	1	0	0	0	1	0
0	0	0	1	0	0	0	1	0
1	1	0	0	0	0	0	2	0
0	1	0	0	0	0	0	1	0
0	1	0	1	0	0	0	2	0
0	0	0	1	0	0	0	1	0
0	1	0	0	0	0	0	1	0
1	0	0	0	0	0	0	1	1
0	1	0	0	0	0	0	1	0
1	1	0	1	0	0	0	3	0
0	1	0	1	0	0	0	2	0
0	1	0	0	0	0	0	1	0
1	0	0	0	0	0	0	1	1
0	1	0	0	0	0	0	1	0
1	0	0	0	0	0	0	1	1
1	0	0	0	0	0	0	1	1

0	1	0	0	0	0	0	1	0
0	1	0	0	0	0	0	1	1
0	1	0	0	0	0	0	1	0
0	1	0	0	0	0	0	1	0
1	0	0	1	0	0	0	2	1
0	0	0	1	0	0	0	1	0
0	1	0	0	0	0	0	1	0
0	1	0	0	0	0	0	1	0
1	1	0	0	0	0	0	2	1
1	1	0	0	0	0	0	2	0
1	0	0	0	0	0	0	1	1
1	1	0	0	0	0	0	2	1
1	0	0	0	0	0	0	1	1
0	1	0	0	0	0	0	1	0
0	0	0	1	0	0	0	1	0
0	1	0	1	0	0	0	2	0
0	1	0	0	0	0	0	1	0
0	1	0	0	0	0	0	1	0
1	1	0	1	0	0	0	3	1
1	1	0	1	0	0	0	3	1
1	1	0	0	0	0	0	2	0
0	1	0	0	0	0	0	1	0
1	1	0	0	0	0	0	2	0
0	1	0	0	0	0	0	1	0
1	1	0	0	0	0	0	2	0
1	0	0	0	0	0	0	1	0
0	1	0	0	0	0	0	1	0
0	1	0	0	0	0	0	1	0
1	1	0	0	1	0	0	3	1
1	1	0	0	0	0	0	2	1
0	0	0	1	0	0	0	1	0
0	1	0	0	0	0	0	1	0
0	0	0	1	0	0	0	1	0
0	0	0	1	0	0	0	1	0
0	0	0	1	0	0	0	1	0
0	0	0	1	0	0	0	1	0
0	1	0	0	0	0	0	1	0
0	1	0	0	0	0	0	1	0
0	1	0	0	0	0	0	1	0
1	1	0	0	0	0	0	2	0
0	0	0	1	0	0	0	1	0
0	1	0	1	0	0	0	2	0
0	1	0	0	0	0	0	1	0
0	1	0	0	0	0	0	1	0
1	1	0	1	0	0	0	3	1
0	1	0	1	0	0	0	2	0
0	1	0	1	0	0	0	2	0
0	1	0	1	0	0	0	2	0
0	1	0	1	0	0	0	2	0
0	1	0	1	0	0	0	2	0

0	1	0	1	0	0	0	2	0
0	1	0	1	0	0	0	2	0
0	1	0	1	0	0	0	2	0
0	1	0	0	0	0	0	1	0
0	1	0	0	0	0	0	1	0
0	1	0	0	0	0	0	1	0
1	0	0	0	0	0	0	1	1
0	0	0	1	0	0	0	1	0
0	1	0	0	0	0	0	1	0
0	1	0	0	0	0	0	1	0
1	0	0	0	0	0	0	1	0
0	1	0	0	0	0	0	1	0
0	1	0	0	0	0	0	1	0
0	1	0	0	0	0	0	1	0
0	1	0	0	0	0	0	1	0
0	1	0	0	0	0	0	1	0
0	1	0	0	0	0	0	1	0
0	1	0	0	0	0	0	1	0
1	0	0	0	0	0	0	1	1
1	0	0	0	0	0	0	1	1
1	0	0	0	0	0	0	1	1
0	1	0	0	0	0	0	1	0
0	1	0	0	0	0	0	1	0
1	0	0	0	0	0	0	1	0
1	0	0	0	0	0	0	1	0
0	1	0	0	0	0	0	1	0
0	1	0	0	0	0	0	1	0
0	1	0	0	0	0	0	1	0
0	1	0	0	0	0	0	1	0
1	1	0	0	0	0	0	2	0
0	1	0	0	0	0	0	1	0
0	1	0	0	0	0	0	1	0
0	1	0	0	0	0	0	1	0
0	1	0	0	0	0	0	1	0
0	1	0	1	0	0	0	2	0
0	0	0	1	1	0	0	2	0
0	1	0	0	0	0	0	1	0
0	1	0	0	1	0	0	2	0
0	1	0	0	0	0	0	1	0
0	1	0	0	0	0	0	1	0
0	1	0	1	0	0	0	2	0
1	0	0	0	0	0	0	1	1
0	0	0	1	0	0	0	1	0
0	1	0	0	0	0	0	1	0
0	1	0	1	0	0	0	2	0
1	1	0	0	0	0	0	2	1
1	1	0	0	0	0	0	2	1
0	1	0	0	0	0	0	1	0
0	1	0	0	0	0	0	1	0
1	1	0	0	0	0	0	2	1

1	0	0	0	0	0	0	1	0
1	1	0	0	0	0	0	2	1
0	1	0	0	0	0	0	1	0
0	1	0	0	0	0	0	1	0
1	0	0	0	0	0	0	1	1
0	1	0	0	0	0	0	1	0
1	0	0	0	0	0	0	1	1
1	1	0	0	0	0	0	2	0
1	0	0	0	0	0	0	1	1
0	1	0	0	1	0	0	2	0
0	1	0	0	0	0	0	1	0
0	1	0	1	0	0	0	2	0
0	1	0	1	0	0	0	2	0
0	0	0	1	0	0	0	1	0
0	0	0	1	0	0	0	1	0
0	1	0	1	0	0	0	2	0
0	1	0	0	0	0	0	1	0
0	1	0	1	0	0	0	2	0
0	1	0	0	0	0	0	1	0
1	0	0	0	0	0	0	1	1
0	1	0	0	0	0	0	1	0
0	1	0	0	0	0	0	1	0
1	0	0	0	0	0	0	1	1
0	1	0	1	0	0	0	2	0
0	1	0	1	0	0	0	2	0
0	1	0	1	0	0	0	2	0
0	1	0	1	0	0	0	2	0
0	0	0	1	0	0	0	1	0
0	0	0	1	0	0	0	1	0
0	1	0	0	0	0	0	1	0
0	1	0	1	0	0	0	2	0
0	1	0	0	0	0	0	1	0
0	1	0	1	0	0	0	2	0
0	1	0	0	0	0	0	1	0
0	0	0	1	0	0	0	1	0
1	0	0	0	0	0	0	1	1
0	1	0	0	0	0	0	1	0
0	1	0	0	0	0	0	1	0
0	1	0	1	0	0	0	2	0
0	0	0	1	0	0	0	1	0
0	1	0	0	0	0	0	1	0
0	1	0	0	0	0	0	1	0
0	1	0	0	0	0	0	1	0
0	1	0	0	0	0	0	1	0
0	0	0	1	0	0	0	1	0
0	1	0	1	0	0	0	2	0
1	1	0	1	0	0	0	3	0
0	1	0	1	0	0	0	2	0
0	1	0	0	0	0	0	1	0
0	1	0	0	0	0	0	1	0

0	1	0	0	0	0	0	1	0
0	1	0	0	0	0	0	1	0
0	1	0	0	0	0	0	1	0
1	1	0	0	0	0	0	2	0
0	1	0	1	0	0	0	2	0
0	1	0	1	0	0	0	2	0
0	1	0	0	0	0	0	1	0
0	1	0	0	0	0	0	1	0
1	0	0	1	0	0	0	2	1
1	0	0	0	0	0	0	1	1
0	1	0	0	0	0	0	1	0
0	1	0	0	0	0	0	1	0
1	1	0	0	0	0	0	2	1
0	1	0	0	0	0	0	1	0
0	1	0	0	0	0	0	1	0
0	1	0	1	0	0	0	2	0
0	1	0	1	0	0	0	2	0
0	0	0	1	0	0	0	1	0
0	1	0	1	0	0	0	2	0
0	1	0	0	0	0	0	1	0
0	1	0	0	0	0	0	1	0
0	0	0	1	0	0	0	1	0
0	1	0	0	0	0	0	1	0
0	1	0	0	0	0	0	1	0
0	1	0	0	0	0	0	1	0
0	1	0	0	0	0	0	1	0
1	1	0	1	1	0	0	4	1
0	0	0	1	1	0	0	2	0
1	1	0	1	0	0	0	3	1
0	1	0	0	0	0	0	1	0
1	1	0	1	0	0	0	3	0
1	1	0	1	0	0	0	3	1
0	1	0	0	0	0	0	1	0
0	1	0	0	0	0	0	1	0
0	1	0	0	0	0	0	1	0
0	1	0	0	0	0	0	1	0
0	1	0	0	0	0	0	1	0
0	1	0	1	0	0	0	2	0
0	1	0	0	0	0	0	1	0
0	1	0	0	0	0	0	1	0
0	1	0	0	0	0	0	1	0
0	1	0	1	0	0	1	3	0
1	1	0	0	0	0	0	2	1
0	0	0	1	0	0	0	1	0
0	1	0	1	0	0	0	2	0
0	1	0	1	0	0	0	2	0
1	1	0	0	0	0	0	2	0
1	0	0	0	0	0	0	1	1
0	1	0	1	0	0	0	2	0
0	1	0	1	0	0	0	2	0

0	1	0	1	0	0	0	2	0
0	1	0	1	0	0	0	2	0
0	1	0	0	0	0	0	1	0
0	1	0	0	0	0	0	1	0
1	1	0	0	0	0	0	2	0
0	1	0	0	0	0	0	1	0
0	0	0	0	1	0	1	2	0
0	0	0	0	0	0	1	1	0
0	1	0	1	0	0	0	2	0
0	1	0	0	0	0	0	1	0
1	1	0	1	0	0	0	3	0
0	1	0	0	0	0	0	1	0
0	1	0	1	0	0	0	2	0
0	1	0	1	0	0	0	2	0
0	1	0	0	0	0	0	1	0
0	1	0	0	0	0	0	1	0
1	0	0	0	0	0	0	1	1
0	1	0	1	0	0	0	2	0
0	1	0	0	0	0	0	1	0
0	1	0	0	0	0	0	1	0
0	1	0	0	0	0	0	1	0
0	1	0	0	0	0	0	1	0
0	1	0	0	0	0	0	1	0
0	1	0	0	0	0	0	1	0
0	1	0	0	0	0	0	1	0
1	1	0	1	1	0	0	4	0
1	1	0	0	0	0	0	2	1
1	1	0	0	0	0	0	2	0
0	1	0	1	0	0	0	2	0
0	1	0	0	0	0	0	1	0
0	1	0	1	0	0	0	2	0
0	1	0	1	0	0	0	2	0
0	1	0	0	0	0	0	1	0
0	1	0	0	0	0	0	1	0
0	1	0	0	0	0	0	1	0
0	1	0	0	0	0	0	1	0
0	1	0	0	0	0	0	1	0
0	1	0	0	0	0	0	1	0
0	1	0	1	0	0	0	2	0
0	1	0	0	0	0	0	1	0
1	1	0	1	0	0	0	3	1
0	1	0	1	0	0	0	2	0
0	1	0	1	0	0	0	2	0
0	1	0	0	0	0	0	1	0
1	1	0	0	0	0	0	2	1
0	1	0	0	0	0	0	1	0
0	1	0	0	0	0	0	1	0
0	1	0	0	0	0	0	1	0
0	1	0	0	0	0	0	1	0
0	1	0	0	0	0	0	1	0

0	1	0	0	1	0	0	2	0
0	1	0	0	0	0	0	1	0
0	1	0	0	0	0	0	1	0
0	1	0	0	0	0	0	1	0
1	1	0	0	0	0	0	2	0
0	1	0	0	0	0	0	1	0
0	1	0	0	0	0	0	1	0
1	1	0	0	0	0	0	2	0
1	1	0	0	0	0	0	2	1
0	1	0	1	0	0	0	2	0
0	1	0	0	0	0	0	1	0
1	1	0	1	0	0	0	3	1
0	1	0	1	0	0	0	2	0
0	1	0	0	0	0	0	1	0
0	1	0	0	0	0	0	1	0
0	1	0	0	0	0	0	1	0
0	1	0	0	0	0	0	1	0
0	1	0	0	0	0	0	1	0
0	1	0	0	0	0	0	1	0
1	1	0	1	0	0	0	3	1
1	1	0	0	0	0	0	2	1
0	1	0	0	0	0	0	1	0
0	1	0	1	0	0	0	2	0
0	1	0	1	0	0	0	2	0
0	0	0	1	0	0	0	1	0
0	1	0	1	0	0	0	2	0
0	1	0	0	0	0	0	1	0
1	0	0	0	0	0	0	1	1
0	1	0	0	0	0	0	1	0
1	1	0	0	0	0	0	2	1
1	0	0	0	0	0	0	1	0
0	1	0	0	0	0	0	1	0
0	1	0	1	0	0	0	2	0
1	1	0	0	0	0	0	2	1
0	0	0	1	0	0	0	1	0
0	0	0	1	0	0	0	1	0
0	1	0	1	0	0	0	2	0
0	1	0	0	0	0	0	1	0
1	1	0	0	0	0	0	2	0
0	1	0	0	0	0	0	1	0
0	0	0	1	0	0	0	1	0
0	0	0	1	0	0	0	1	0
0	1	0	0	0	0	0	1	0
0	1	0	0	0	0	0	1	0
0	1	0	0	0	0	0	1	0
0	1	0	0	0	0	0	1	0
0	1	0	0	0	0	0	1	0
0	1	0	1	0	0	0	2	0
0	1	0	0	0	0	0	1	0
0	1	0	0	0	0	0	1	0

0	1	0	1	0	0	0	2	0
0	1	0	0	0	0	0	1	0
0	1	0	0	0	0	0	1	0
0	1	0	0	0	0	0	1	0
1	1	0	0	0	0	1	3	1
0	1	0	1	0	0	0	2	0
0	1	0	0	0	0	0	1	0
0	1	0	0	0	0	0	1	0
0	1	0	0	0	0	0	1	0
0	1	0	0	0	0	0	1	0
0	1	0	0	0	0	0	1	0
1	1	0	0	0	0	0	2	1
1	1	0	0	0	0	0	2	1
0	1	0	0	0	0	0	1	0
0	1	0	0	0	0	0	1	0
0	1	0	1	0	0	0	2	0
0	1	0	0	0	0	0	1	0
0	0	0	1	0	0	0	1	0
0	0	0	1	0	0	0	1	0
1	0	0	0	0	0	0	1	0
1	1	0	0	0	0	0	2	1
1	0	0	1	0	0	0	2	1
1	1	0	1	0	0	0	3	0
0	1	0	0	0	0	0	1	0
1	1	0	0	0	0	0	2	1
0	1	0	0	0	0	0	1	0
0	1	0	0	0	0	0	1	0
0	1	0	0	0	0	0	1	0
0	1	0	0	0	0	0	1	0
0	1	0	0	0	0	0	1	0
0	1	0	0	0	0	0	1	0
0	1	0	0	0	0	0	1	0
0	1	0	0	0	0	0	1	0
0	1	0	0	0	0	0	1	0
1	1	0	0	0	0	1	3	0
0	1	0	0	0	0	0	1	0
0	0	0	1	0	0	0	1	0
0	1	0	0	0	0	0	1	0
0	1	0	0	0	0	0	1	0
0	1	0	1	0	0	0	2	0
0	1	0	0	0	0	0	1	0
1	1	0	0	0	0	0	2	1
1	1	0	1	0	0	0	3	1
0	0	0	1	0	0	0	1	0
1	0	0	0	0	0	0	1	1
1	0	0	0	0	0	0	1	1
0	1	0	0	0	0	0	1	0
0	1	0	0	0	0	0	1	0
0	0	0	1	0	0	0	1	0
0	1	0	0	0	0	0	1	0

0	1	0	0	0	0	0	1	0
0	0	0	1	0	0	0	1	0
0	0	0	0	1	0	0	1	0
0	1	0	0	0	0	0	1	0
0	0	0	1	0	0	0	1	0
0	0	0	1	0	0	0	1	0
0	0	0	1	0	0	0	1	0
0	0	0	1	0	0	0	1	0
1	0	0	0	0	0	0	1	1
0	0	0	1	0	0	0	1	0
1	1	0	0	0	0	0	2	1
0	0	0	1	0	0	0	1	0
0	1	0	1	0	0	0	2	0
0	1	0	0	0	0	0	1	0
0	1	0	0	0	0	0	1	0
0	1	0	0	0	0	0	1	0
0	1	0	1	0	0	0	2	0
0	1	0	1	0	0	0	2	0
0	1	0	1	0	0	0	2	0
1	0	0	0	0	0	0	1	0
0	1	0	0	0	0	0	1	0
0	0	0	1	0	0	0	1	0
0	1	0	1	0	0	0	2	0
1	1	0	0	0	0	0	2	0
1	0	0	0	0	0	0	1	0
0	0	0	1	0	0	0	1	0
0	1	0	1	0	0	0	2	0
0	0	0	1	0	0	0	1	0
0	0	0	1	0	0	0	1	0
0	0	0	1	0	0	0	1	0
0	1	0	1	0	0	0	2	0
0	1	0	0	0	0	0	1	0
0	1	0	0	0	0	0	1	0
0	1	0	0	0	0	0	1	0
1	0	0	0	0	0	0	1	0
0	0	0	0	1	0	0	1	0
0	0	0	0	1	0	0	1	1
0	0	0	0	1	0	0	1	1
0	0	0	0	1	0	0	1	0
1	1	0	0	0	0	0	2	0
1	1	0	0	0	0	0	2	1
0	1	0	0	0	0	0	1	0
1	1	0	0	0	0	0	2	0
0	1	0	1	0	0	0	2	0
0	1	0	1	0	0	0	2	0
0	1	0	1	0	0	0	2	1
1	1	0	0	0	0	0	2	1
1	1	0	0	0	0	0	2	0
1	0	0	0	0	0	0	1	1
0	1	0	0	0	0	0	1	0

0	0	0	1	0	0	0	1	0
0	1	0	1	0	0	0	2	0
0	1	0	1	0	0	0	2	0
0	1	0	1	0	0	0	2	0
0	1	0	1	0	0	0	2	0
1	1	0	0	0	0	0	2	1
0	1	0	0	0	0	0	1	0
0	1	0	0	0	0	0	1	0
0	1	0	0	0	0	0	1	0
0	1	0	1	0	0	0	2	0
1	1	0	1	0	0	0	3	0
0	1	0	1	0	0	0	2	0
0	1	0	1	0	0	0	2	0
1	1	0	0	0	0	0	2	1
1	1	0	1	0	0	0	3	1
1	1	0	1	0	0	0	3	1
1	1	0	1	0	0	1	4	1
1	0	0	0	0	0	0	1	0
1	0	0	0	0	0	0	1	1
0	0	0	1	0	0	0	1	0
0	0	0	1	0	0	0	1	0
0	1	0	0	0	0	0	1	0
0	0	0	0	0	0	0	0	0
0	1	0	0	0	0	0	1	0
0	1	0	0	0	0	0	1	0
1	1	0	1	0	0	0	3	1
0	1	0	1	0	0	0	2	0
0	1	0	1	0	0	0	2	0
1	1	0	0	0	0	0	2	0
1	1	0	0	0	0	0	2	0
0	1	0	0	0	0	0	1	0
0	1	0	0	0	0	0	1	0
0	1	0	0	0	0	0	1	0
0	1	0	0	0	0	0	1	0
0	1	0	0	0	0	0	1	0
0	1	0	0	0	0	0	1	0
1	1	0	0	0	0	0	2	0
1	1	0	0	0	0	0	2	0
0	1	0	0	0	0	0	1	0
0	1	0	0	0	0	0	1	0
0	1	0	1	0	0	0	2	0
0	1	0	1	0	0	0	2	0
0	1	0	0	0	0	0	1	0
0	1	0	0	0	0	0	1	0
1	0	0	0	0	0	0	1	1
0	1	0	0	0	0	0	1	0
0	1	0	0	0	0	0	1	0
0	1	0	0	0	0	0	1	0
0	1	0	1	0	0	0	2	0
0	1	0	0	0	0	0	1	0

0	1	0	1	0	0	0	2	0
